# Magnetic Interactions in a [Co(II)_3_Er(III)(OR)_4_] Model Cubane through Forefront Multiconfigurational Methods

**DOI:** 10.1021/acs.jctc.2c01318

**Published:** 2023-05-01

**Authors:** Ruocheng Han, Sandra Luber, Giovanni Li Manni

**Affiliations:** † Department of Chemistry A, University of Zurich, Winterthurerstrasse 190, 8057 Zurich, Switzerland; ‡ Max Planck Institute for Solid State Research, Heisenbergstrasse 1, 70569 Stuttgart, Germany

## Abstract

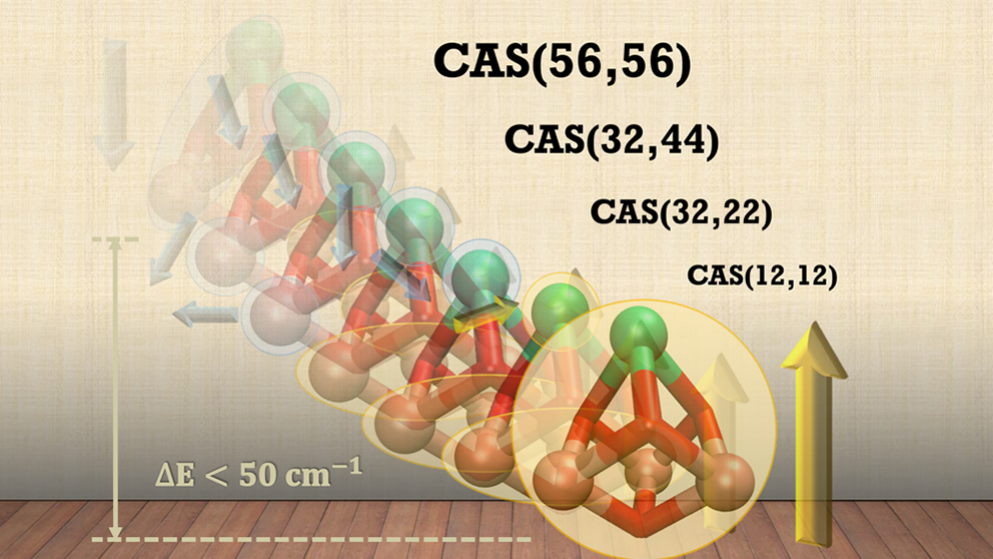

Strong electron correlation effects are one of the major challenges in modern quantum chemistry. Polynuclear transition metal clusters are peculiar examples of systems featuring such forms of electron correlation. Multireference strategies, often based on but not limited to the concept of complete active space, are adopted to accurately account for strong electron correlation and to resolve their complex electronic structures. However, transition metal clusters already containing four magnetic centers with multiple unpaired electrons make conventional active space based strategies prohibitively expensive, due to their unfavorable scaling with the size of the active space. In this work, forefront techniques, such as density matrix renormalization group (DMRG), full configuration interaction quantum Monte Carlo (FCIQMC), and multiconfiguration pair-density functional theory (MCPDFT), are employed to overcome the computational limitation of conventional multireference approaches and to accurately investigate the magnetic interactions taking place in a [Co(II)_3_Er(III)(OR)_4_] (chemical formula [Co(II)_3_Er(III)(hmp)_4_(μ_2_-OAc)_2_(OH)_3_(H_2_O)], hmp = 2-(hydroxymethyl)-pyridine) model cubane water oxidation catalyst. Complete active spaces with up to 56 electrons in 56 orbitals have been constructed for the seven energetically lowest different spin states. Relative energies, local spin, and spin–spin correlation values are reported and provide crucial insights on the spin interactions for this model system, pivotal in the rationalization of the catalytic activity of this system in the water-splitting reaction. A ferromagnetic ground state is found with a very small, ∼50 cm^–1^, highest-to-lowest spin gap. Moreover, for the energetically lowest states, *S* = 3–6, the three Co(II) sites exhibit parallel aligned spins, and for the lower states, *S* = 0–2, two Co(II) sites retain strong parallel spin alignment.

## Introduction

Understanding the role of strong electron correlation in systems with complex electronic structures toward their spectroscopic and catalytic properties is one of the major challenges in modern quantum chemistry. The family of polynuclear transition-metal (PNTM) clusters represents a pivotal example of such systems, with their magnetic properties directly dependent on electron correlation effects, that in turn strongly affect their catalytic behavior. The structures computationally investigated to date, such as [Fe_2_S_2_],^1−4^ [Fe_4_S_4_],^2−5^ [Fe_8_S_7_],^6^ [Sr_2_CuO_3_] and [La_2_CuO_4_] cuprates,^7^ [Mn_2_O_2_],^8−10^ [Mn_3_O_4_],^11−13^ and [Mn_4_CaO_5_],^13,14^ are characterized by magnetic centers featuring a variable number of unpaired 3d electrons, bridged with oxygen or sulfur ligand atoms. Electron correlation effects, in the form of direct-exchange, superexchange, charge-transfer, and correlated orbital relaxations affect the magnetic interactions across the metal centers. From a computational standpoint, very few quantum chemistry methods are able to qualitatively correctly take into account such correlation effects, making any prediction about relative stability of the low-lying electronic states difficult and only possible via highly specialized and computationally demanding methodologies that use multireference wave function ansätze for the characterization of the electronic states of interest. Moreover, the energy gap between the highest and the lowest spin state or between other spin states of interest is often only hundreds of cm^–1^, making it computationally challenging to quantitatively accurately predict the ground state electronic structure and relevant spin gaps. While full configuration interaction (FCI) can provide exact solutions for such demanding electronic problems, the exponential scaling of the method with respect to the number of basis functions prevents its usage in practical applications. Methods based on the concept of active space (AS) have been introduced to reduce the computational costs associated with the FCI method, by allowing a truncation of the FCI many body wave function, based on chemical/physical arguments. Active space based approaches include the complete active space (CAS) strategy,^15^ in its conventional^16−20^ and stochastic^2,7,21−24^ forms, or driven by a density matrix renormalization group (DMRG) many-body optimizer,^25−41^ the restricted active space (RAS) approach,^20,42,43^ the occupation restricted multiple active spaces (ORMAS) method,^44^ and the generalized active space (GAS) strategy, in both its deterministic^45,46^ and stochastic^47,48^ forms. In active space approaches, a set of electrons and orbitals around and including the *frontier orbitals* (HOMO and LUMO) are chosen to be *active*, and a multiconfigurational space is built by exciting in all possible ways (fulfilling space and spin symmetry conditions) the active electrons among the active orbitals. Complete active spaces are generally labeled as CAS(*X*,*Y*) where *X* and *Y* represent the number of active electrons and orbitals, respectively. In RAS and GAS, constraints are imposed by dividing the active space into active subspaces and limiting the level of interspace excitations. The orbitals outside the active space can further be variationally optimized via a self-consistent field (SCF) procedure, that leads to the CASSCF, RASSCF, and GASSCF approaches. The variational relaxation of orbitals under the mean-field generated by the multiconfigurational wave function is pivotal, as it removes any bias with respect to the initial trial wave function. The CI problem within the active space is solved either with exact diagonalization (Jacobi),^49^ via iterative procedures based on the power method (Davidson, Lanczos),^50−52^ or with strategies that approximate the exact eigensolutions, such as the density matrix renormalization group approach (DMRG)^26^ and full configuration interaction quantum Monte Carlo (FCIQMC).^53−57^ The DMRG approach has been widely and reliably adopted for studying transition metal clusters.^3,6,8,10,14,58,59^ In recent years, Li Manni and co-workers have demonstrated the successful application of stochastic approaches, in the form of CAS and GAS schemes,^2,21,48^ onto single-metal^22,23^ and PNTM complexes.^1,2,5,7,11,12^ In this context, of particular interest is the wave function compression that arises when a spin adapted many body basis is utilized, such as in the graphical unitary group approach (GUGA) based approaches, and particular unitary transformations of the molecular orbitals are performed, in the form of localizations and reorderings.^1,2,5,11,60,61^ Under these conditions, very compact optimized many body wave functions are obtained, to the limit of single reference wave functions, a property that is extremely beneficial to FCIQMC and related stochastic methods and to those methods that take advantage of the sparsity of the Hamiltonian matrix and its eigensolutions.

In this study, the electronic structure of the energetically low-lying states of a [Co(II)_3_Er(III)(OR)_4_] cluster model is investigated in great detail via the forefront Stochastic-CAS, DMRG, and MC-PDFT techniques and using increasingly larger active space many body wave functions.

[Co(II)_3_Er(III)(OR)_4_] is one of the closest mimics for the photosystem II (PSII) oxygen evolving complex also being active for water oxidation.^62^ To date, this system has only been investigated at the density functional theory (DFT) level of theory,^62,63^ with main focus on the geometric and energetic properties of the cubane structure in water and possible water oxidation mechanisms, while its electronic structure remains to a large extent uncharted, inhibiting the understanding of the electron transfer process and further design of water-oxidizing complexes (WOCs). As in other PNTM clusters, weak exchange-coupled magnetic interactions among the metal centers, mediated by the bridging oxygen atoms and susceptible to spin-frustration effects, result at the quantum chemical level in wave functions with substantial multireference character, which can hardly be accurately described using DFT based calculations.

We have applied wave function based methods, including Stochastic-CASSCF and DMRG-CASSCF for multiple choices of increasingly larger active spaces, up to CAS(56,56), which includes all singly and doubly occupied 3d or 4f orbitals of the metal centers, the double-shell orbitals, and the 2p orbitals of the bridging atoms and their electrons. Complete active space second-order perturbation theory, CASPT2^64,65^ (only for small active space reference wave functions), and multiconfiguration pair-density functional theory, MCPDFT^66^ (also possible in combination with large active space wave functions), have been utilized to account for dynamic correlation effects.

Our results indicate that all energetically low electronic states computed in this work are nearly degenerate, with lowest-to-highest spin-gap on the order of 30–50 cm^–1^, which matches well its behavior as catalyst, as suggested in the literature.^67,68^

The local spin and spin–spin correlation values, calculated on the basis of the large CAS(56,56) stochastic computations, provide easy and intuitive ways to interpret the physics of the intramolecular magnetic interactions along the spin-ladder, of crucial importance in the rationalization of the catalytic mechanism in which this cluster is involved.

Experimentally, the [Co(II)_3_Er(III)(OR)_4_] cubane has been found to be the most active water oxidation catalyst in an entire series of Ln-containing cubanes [Co_3_Ln(OR)_4_], Ln = Ho–Y.^62^ The investigated [Co(II)_3_Er(III)(OR)_4_] model has also successfully been used in computational studies for water oxidation mechanisms and factors influencing the catalytic behavior.^63,68^ We identified various aspects supporting efficient catalytic behavior such as flexibility of the catalyst with respect to ligand environment, charge, protonation states, and nuclear structure,^69^ even leading to the discovery of open cubane structures in close analogy to the oxygen-evolving complex in photosystem II.^63^ Moreover, it has become apparent that spin states play an important role and can make a significant difference in the energetics of the water oxidation mechanism.^68−70^ Energetically close-lying electronic states also can contribute positively to increased flexibility during catalysis, allowing, e.g., switching between different potential energy surfaces depending on the need for efficient catalysis, also in combination with changes in nuclear structure.

## Computational and Theoretical Details

### [Co(II)_3_Er(III)(OR)_4_] Cubane Structure

**Figure 1:**
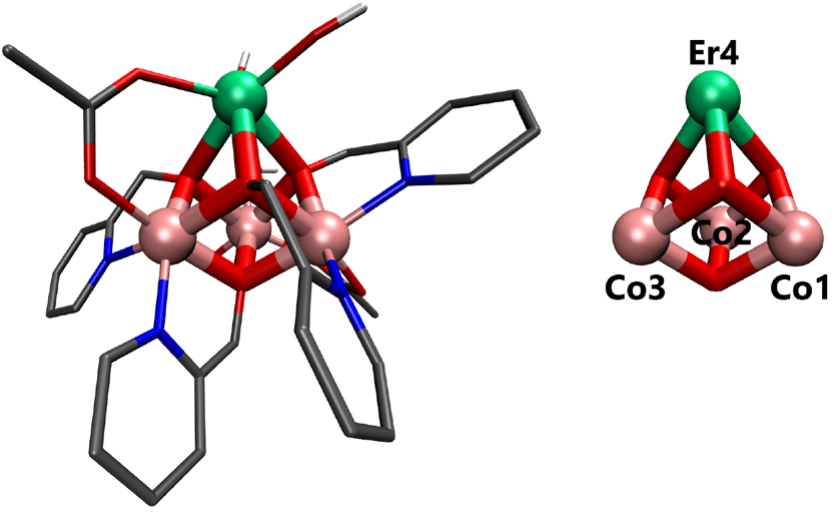
Nuclear structure of [Co(II)_3_Er(III)(OR)_4_] cubane. Hydrogen atoms are omitted for convenience (except when attached to oxygen). Pink: cobalt, green: erbium, red: oxygen, blue: nitrogen, gray: carbon, white: hydrogen.

The [Co(II)_3_Er(III)(OR)_4_] cubane structure employed in the present work is taken from a previous work,^63^ in which DFT-based geometry optimizations and molecular dynamics with explicit solvent have been utilized to explore the catalytic cycle (see Figure 1). The core structure of the cubane contains one Er(III) and three Co(II) transition metal ions and four bridging oxygen atoms.^62^ The bridging oxygens are the terminal atoms of deprotonated 2-(hydroxymethyl)pyridine moieties. The net charge of the whole system is zero. The Cartesian coordinates for the model system investigated are provided in the Supporting Information. The valence electronic configuration is 3d^7^ for Co(II) and 4f^11^ for Er(III). Locally, each metal center experiences a quasi-octahedral ligand field, with each metal ion surrounded by six ligand groups. There are 3 unpaired electrons on each site, for a total of 12 unpaired electrons. As we show later in this work via computations, for the energetically lowest spin states, the second Hund’s rule (maximal spin) applies for each individual metal center, *S*_loc_ = 3/2, and the four 3/2-spins may couple in seven different total spin rearrangements, with total spin *S*_tot_ = 0–6. Combining two local spins with *S*_loc_ = 3/2 results in 4 intermediate spin states with *S*_interm_ = 0–3. These intermediate states can further combine with a third *S*_loc_ = 3/2 local spin, leading to 12 three-center intermediate states (see, for example, eq 2 of ref (11)). The 12 intermediate three-center spin states will further spin couple to the last magnetic center with *S*_loc_ = 3/2, leading to one *S*_tot_ = 6, three *S*_tot_ = 5, six *S*_tot_ = 4, ten *S*_tot_ = 3, 11 *S*_tot_ = 2, nine *S*_tot_ = 1, and four *S*_tot_ = 0. Thus, a total of 44 spin states characterize the low-energy spectrum of this four-center model system.

### Active Space Models

In the CAS approach, the orbital space is divided into three parts: the *inactive* orbitals are doubly occupied in all electronic configurations of the CI expansion; the *active* orbitals represent the orbital space in which the CI expansion is generated; the *virtual* orbitals are kept empty in all electronic configurations of the CI expansion. In general orbitals responsible for strong electron correlation are chosen to be active. Those include singly occupied orbitals or doubly occupied/empty orbitals that play a key role in terms of strong electron correlation effects. If the CI problem is solved on a fixed molecular orbital basis (say the Hartree–Fock orbitals), we use the CASCI label. This approach carries the bias of the initial orbital set. This topic has been discussed in detail in earlier works by one of the authors.^2^ When the orbitals are variationally optimized using an SCF procedure, under the mean-field generated by the multiconfigurational wave function within the active space, we use the CASSCF label. Both CASCI and CASSCF wave function ansätze have been adopted in this work.

**Figure 2:**
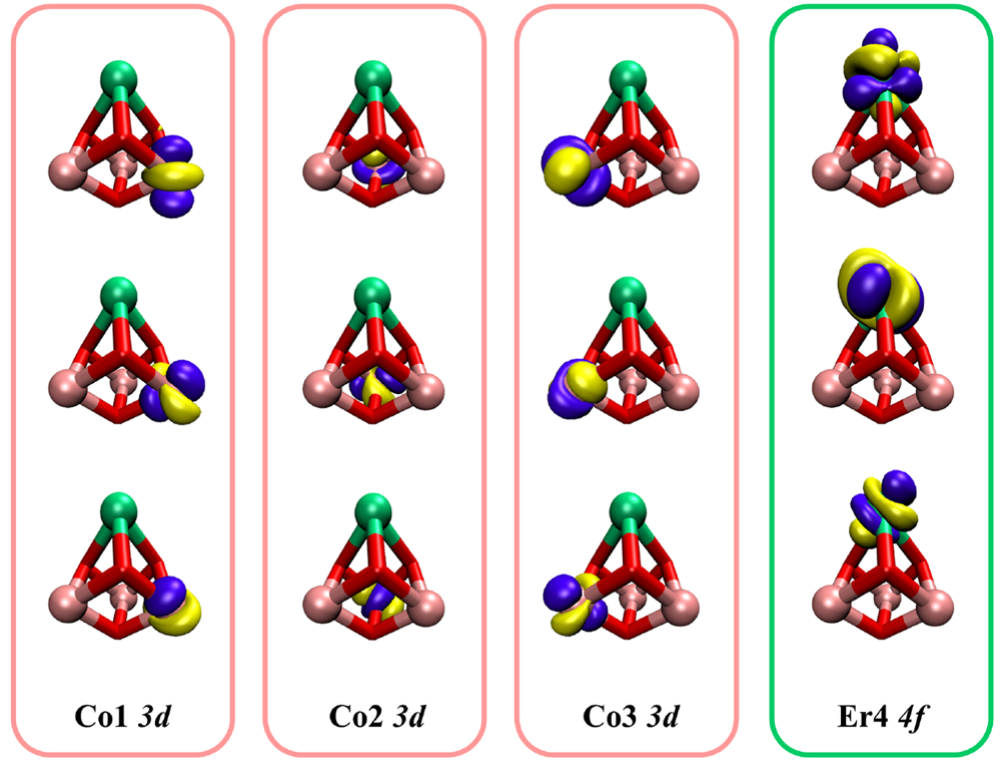
Twelve orbitals selected in CAS(12,12) calculations. Only the core structure of the cubane is shown. Pink: cobalt, green: erbium, red: oxygen.

The smallest active space conceivable is the CAS(12,12) (see orbitals in Figure 2), which consists of the 12 singly occupied orbitals and their 12 unpaired electrons (3 singly occupied 3d orbitals of each Co^(II)^ ion and 3 singly occupied 4f orbitals of the Er^(III)^ ion). Electron correlation effects arising from the interactions between unpaired and paired electrons within the same magnetic site have been considered in the larger CAS(32,22), consisting of the valence 3d and 4f orbitals and their electrons. The double-shell effect (e.g., see refs (22 and 71)), which describes radial electron correlation^72^ at the metal centers, has been considered in the next larger model active space, CAS(32,44), where the empty correlating d′ (or 4d) orbitals on the Co ions and the correlating f′ (or 5f) orbitals on the Er ion have been added to the valence-only (32,22) active space. Correlation effects related to the O–M hopping mechanisms (charge-transfer, superexchange) are explicitly considered in the CAS(56,56) that also includes the 24 2p electrons of the four bridging oxygen atoms and their 12 orbitals. The latter represents the largest active space utilized in this work.

The bigger the active space, the exponentially larger the CI expansion. In practice, conventional diagonalization techniques (Davidson) can be applied for active spaces that contain at most ∼18 electrons and ∼18 orbitals, CAS(18,18). For large active spaces, only approximated or truncated CI eigensolvers can be adopted, such as DMRG, FCIQMC, GAS or selected-CI schemes. Correlation effects outside the active space are commonly described via perturbation theory approaches, such as CASPT2^64,65^ or, more recently, via the computationally inexpensive MCPDFT approach.^66,73−75^ Notably, in this work CASPT2 has only been coupled to the small CAS(12,12) wave functions, for which the conventional CASSCF(12,12) procedure and related higher order density matrices are available. For larger active space calculations higher-order density matrices are currently unavailable for practical calculations, thus limiting the applicability of PT2 approaches to large active space reference wave functions. On the contrary, MCPDFT only relies on two-body reduced density matrices, which are easy to calculate both in stochastic and in DMRG methods.

### Procedure and Computational Settings

In the following, the protocol employed to carry out the calculations is outlined:

- **Starting orbitals from the highest spin state (*S*_tot_ = 6)**. The starting orbitals have been obtained from the CASSCF(12,12) on the highest (*S*_tot_ = 6) spin state. This active space choice results in the single configurational trial wave function for the high spin state, |*uuu*, *uuu*, *uuu*, *uuu*⟩ (*u* and *d* labels indicate cumulative spin-up and spin-down spin couplings, respectively^15^). The CAS(12,12) is the smallest active space that allows explicit correlation and spin-coupling of the 12 unpaired electrons for all spin states investigated. The optimized CASSCF(12,12) orbitals of the highest spin state have been localized using the Pipek–Mezey localization procedure^76^ and site-reordered, first by listing the 3d orbitals of Co1, then the 3d orbitals of Co2 and Co3, and finally the 3d orbitals of Er4 (see Figure 1). The localization and reordering process is important because it leads to an extremely sparse electronic Hamiltonian and highly compressed eigenstates of easier physical rationalization. Interested readers will find further details in the literature.^1,2,5,11,60,61^
- **CASSCF(12,12) calculations for*S*_tot_= 1–5 spin states.** The localized and reordered CASSCF(12,12) orbitals of a higher spin state (*S*_tot_ = *s*, *s* = 1–6) are utilized as starting orbitals for the next lower spin state (*S*_tot_ = *s* – 1). This stepwise procedure ensures minimal changes in the orbital relaxation step (SCF), and it facilitates stability and fast convergence in the CASSCF procedure. A comparison of CASSCF(12,12) energies with and without the stepwise optimization procedure is offered in the Supporting Information (CASSCF(12,12) optimization procedure and Table S1). At this level of theory, we have calculated one *S*_tot_ = 6, three *S*_tot_ = 5, six *S*_tot_ = 4, ten *S*_tot_ = 3, 11 *S*_tot_ = 2, nine *S*_tot_ = 1, and four *S*_tot_ = 0, which form the entire set of low-energy multiplets for this system (as discussed above).
- **CASPT2 and MCPDFT calculations.** The optimized CASSCF(12,12) wave functions from the above steps are coupled to the subsequent CASPT2 and MCPDFT procedures, necessary for recovering dynamic electron correlation effects beyond CASSCF. Two PT2 schemes were adopted. In the CASSCF(12,12)//PT2(full) scheme the entire (*full*) orbital space has been correlated at the PT2 level, except for the 77 energetically lowest core orbitals (see Table S2). Moreover, the frozen natural orbital (FNO) approximation was adopted, and only 501 virtual orbitals have been correlated for the CASSCF(12,12)//PT2(full) scheme. An ionization potential–electron affinity (IPEA) shift^77^ value of 0.25 au was chosen for all CASPT2 calculations. It is worth noting that whether and how the IPEA shift influences the PT2 results is still investigated.^78−81^ Test calculations for the choice of the FNO threshold and for different IPEA shift parameters are summarized in Tables S3 and S4. An FNO threshold of 0.7 units is the largest truncation with a marginal effect on total energies. Smaller threshold values (bigger truncation) showed a substantial impact on spin gap predictions. As already documented in the literature, the IPEA shift has a nonconverging effect on the spin gap, and in absence of reference, the default value has been chosen. MCPDFT calculations, using tPBE and tBLYP (the prefix “t” is used to denote “translated”) functionals,^66^ have been performed to complement and compare against the CASSCF(12,12)//PT2(full) scheme. The CASPT2 and MCPDFT predictions provide an initial estimate of the energetic trend across the spin states investigated that will be discussed in greater detail in the Results and Discussion section. In the second CASSCF(12,12)//PT2(*X*,*Y*) scheme, the PT2 method is employed to selectively explore precise forms of electron correlation outside the CASSCF(12,12) space and inside the larger CAS(*X*,*Y*). This strategy, already used in other contexts,^11,22^ can be understood as an approximate method to recover part of the correlation in the larger CAS(*X*,*Y*), relying on the CASSCF(12,12) reference wave function, while ignoring any orbital relaxation effect and higher order excitation effects. This approach is generally faster but less accurate than DMRG or FCIQMC simulations within the same (*X*,*Y*) active space. Besides providing some insights on the differential effects of the various forms of electron correlations with respect to the relative stability of the different spin states investigated, this approach also provides the means to understand the computational limitations of the second-order perturbation theory, by comparing the PT2(*X*,*Y*) predictions to the more accurate FCIQMC and DMRG calculations on the same CAS(*X*,*Y*) space, specifically the CAS(56,56) model active space. The choice of the CAS(*X*,*Y*) is discussed in detail in the following. We have also carried outsome preliminary calculations at the CAS(12,12)PT2 and at the CAS(12,12)MCPDFT level for all 44 states of the spin multiplet set.
- **Construction of the larger (*X*,*Y*) active spaces.** CAS(32,22), CAS(32,44), and CAS(56,56) spaces have been constructed starting from the variationally relaxed CASSCF(12,12) orbitals of each spin state. To that end, the inactive and virtual orbitals have been localized separately (a procedure that leaves the CASSCF(12,12) energetically unchanged, i.e., *invariant transformations*), and the relevant doubly occupied 3d and 4f orbitals, as well as the double-shell and bridging orbitals, have been selected to be active together with the localized initial 12 active orbitals. These orbitals were used as the basis in the larger CASCI calculations, using DMRG and FCIQMC techniques, and in the CASSCF(12,12)//PT2(*X*,*Y*) approach.
- **Reordering of orbitals for optimal wave function optimization.** Optimal orbital orderings have been found to be essential both in DMRG^29,35−41^ and GUGA-FCIQMC^1,2,5,11^ for stable and fast converging calculations with respect to the internal parameters of the methods, namely, the bond dimension, *M*, in DMRG and the walker number in FCIQMC. DMRG and FCIQMC CI calculations on exchange-coupled magnetic systems using nonlocalized and non-reordered orbitals often cause severe convergence problems^1^ (see also Table S5). It has been widely recognized that the orbital ordering is of pivotal importance for the fast convergence of DMRG optimizations with respect to the bond dimension parameter, *M*.^29,35−41^ Li Manni and co-workers have found that the orbital ordering has a pivotal impact toward more compact GUGA spin-adapted wave function representations, which in turn affect the speed of convergence of approximated eigensolvers, such as the stochastic GUGA-FCIQMC algorithm, as the method takes advantage of the sparsity of the CI Hamiltonian and its eigensolutions. In this respect the localized orbitals of the largest CAS(56,56) have been reordered to achieve maximum compression. In previous works,^1,5,11^ a general rule for best wave function compressions within the GUGA framework was suggested, and it was found beneficial to aggregate the unpaired electrons in an atom-separated manner. Moreover, site permutational searches have been carried out and permutational search algorithms advised,^60^ in order to obtain the best site-ordering. In the previous studies on PNTM clusters,^1,2,5,11^ the optimal ordering could be chosen on the basis of chemical/physical arguments that reflected the internal symmetries of the complex. For example in the case of the FeS cubanes two *spin pairs* were identified and the ordering was chosen to keep the magnetic sites of each pair in consecutive ordering. More recently, a connection has been identified between the block diagonal structure of the CI Hamiltonians of exchange-coupled spin systems and commutation relations between cumulative partial spin operators and the Hamiltonian operator *Ĥ*.^61^ The reduced point group symmetry of the [Co(II)_3_Er(III)(OR)_4_] cubane does not allow easy identification of optimal ordering based solely on symmetry arguments, instead a systematic site permutational search was carried out on the basis of the small CAS(12,12) singlet wave function (*S*_tot_ = 0). The localized CASSCF(12,12) singlet active orbitals were grouped per site (three 3d orbitals for each of the Co1, Co2, and Co3 sites and three 4f orbitals of the Er4 site). A permutational space of 4! site configurations was generated. Within the GUGA formalism and using a conventional Davidson driven CI eigensolver, the CASCI(12,12) singlet wave function was optimized, and the weight of the dominant CSF was used as a parameter to judge the compactness of the wave function, as a simpler alternative to the *L*_1_ and *L*_4_ norms used in previous works.^1,61^ The site-permutation with the most compact CAS(12,12) singlet wave function was then adopted for all larger active space GUGA-FCIQMC simulations. Within the DMRG approach the ordering that led to the fastest convergence with respect to the *M* parameter was chosen as optimal for each model active space here investigated. We wish to stress at this point that the reorderings in DMRG and in GUGA are motivated by fundamentally different arguments, as already discussed in previous works;^5,60^ thus, the best reordering that compresses the many-body wave function is not necessarily the best reordering for a fast converging DMRG procedure with respect to *M*. The Fiedler^83^ and the genetic algorithm (GA)^35^ were utilized, and four different orderings were tested within the DMRG framework.
- **DMRGCI and FCIQMC CASCI(56,56) Calculations.** DMRGCI calculations with bond dimension values, *M*, up to *M* = 5000 and FCIQMC calculations with up to 200 × 10^6^ walkers were carried out. The reference configuration state functions (CSFs) for the GUGA-FCIQMC(56,56) calculations are chosen based on the dominant CSF in the corresponding CASCI(12,12) calculations of each spin state on the localized orbital basis. We would like to stress that no attempt was made to optimize the FCIQMC(56,56) wave functions for all 44 spin multiplet states. These computations will be presented in a future publication with more focus on the prediction of magnetic coupling coefficients and magnetic susceptibility.

### Additional Computational Details

The OpenMolcas^84^ package was employed for all CASSCF, CASPT2, and MCPDFT calculations, and for generating the integral files (FCIDUMP) for the large active space FCIQMC and DMRG calculations.

Atomic natural orbital-relativistic core-correlated basis sets with double-ζ plus polarization quality (ANO-RCC-VDZP) were used for all calculations. The contraction of primitive functions for each atom is Co(21s,15p,10d,6p → 5s,4p,2d,1f), Er(25s,22p,15d,11f,4g → 7s,6p,4d,2f,1g), C,N,O(14s,9p,4d → 3s,2p,1d), and H(8s,4p → 2s,1p), leading to a total of 961 basis functions. Scalar relativistic effects were introduced via second order Douglas–Kroll–Hess integral correction. Cholesky decomposition technique^84,85^ was applied to simplify the computation of the electron repulsion integrals, with a threshold of 10^–3^ au. Orbital localizations were carried out using the Pipek–Mezey localization procedure.^76^

For the CAS(12,12) calculations, the dimension of the explicit Hamiltonian used as preconditioner for the Davidson procedure was set to 1000 for *S*_tot_ = 1–6 and to 10000 for *S*_tot_ = 0. The latter choice was necessary for improving speed of convergence of the Davidson diagonalization procedure. In CASPT2 calculations, an imaginary shift^86^ of 0.2 au and IPEA shift of 0.25 au were utilized for all PT2 calculations, except for the comparisons of different IPEA values (0.10, 0.25, 0.40) in Table S4.

The DMRGCI(56,56) calculations with *M* = 1000, 2000, 3000, 4000, and 5000 were carried out step-by-step for *S*_tot_ = 0, 3, and 6 spin states using the BLOCK code^29,30,87^ (version 1.5.3). The input examples of DMRGCI calculations are provided in section S3. The GUGA-FCIQMC(56,56) calculations with up to 200 × 10^6^ walkers were carried out for *S*_tot_ = 0, 3, and 6 spin states using the NECI code (stable branch of the developer version).^24^ The input examples of FCIQMC calculations are provided in section S4.

## Results and Discussion

### Localization and Reordering of Active Orbitals

**Figure 3:**
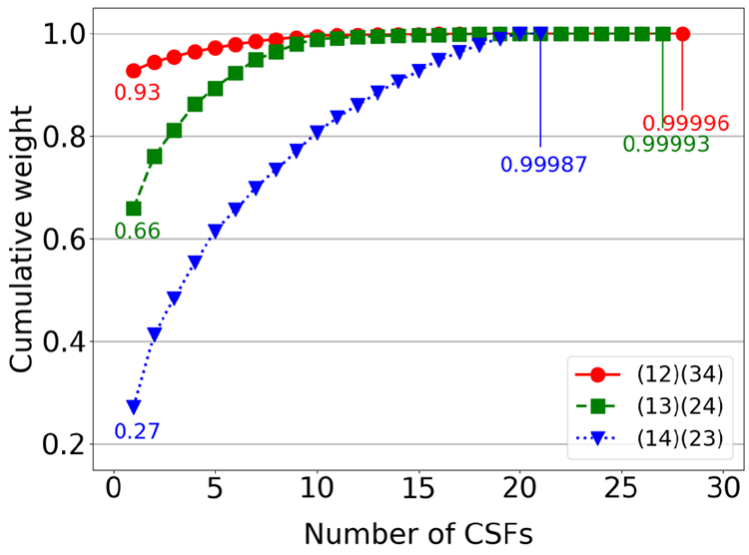
Partial *L*_2_ norm over the CSFs ordered by decreasing weight, for the CASCI(12,12) singlet wave function and using the three ordering types, (12)(34), (13)(24), and (14)(23).

Via a systematic permutational search, of the 4! possible site permutations, only three distinct site-reorderings have been identified, that we have summarized in Table 1 together with the leading CSF and its weight, *C*_*i*_^2^. The orderings within each of the three distinct permutation types share the same wave function structure and thus compression level. For each ordering type, an 8-fold permutational symmetry emerges:
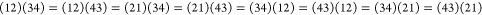
1The dominant CSF in the (12)(34) ordering type has the largest weight. The partial *L*_2_ norm over the CSFs ordered by decreasing weight is illustrated in Figure 3. The partial *L*_2_ norm shows that the (12)(34) ordering type is quickly converging to its extreme value of 1.0 (for fully *L*_2_-normalized wave functions), as compared to the (13)(24) and (14)(23) permutation types.

**Table 1:** Dominant CSF and Its Weight, *C*_*i*_^2^, for the CASCI(12,12) Singlet Wave Functions as a Function of the Site Permutations^a^

orderings	ordering type	dominant spin configuration and its weight
1234, 1243, 2134, 2143 3412, 4312, 3421, 4321	(12)(34)	*uuuuuudddddd* 0.92808
1324, 1342, 3124, 3142 2413, 4213, 2431, 4231	(13)(24)	*uuuddduuuddd* 0.65949
1423, 1432, 4123, 4132 2314, 3214, 3241, 3241	(14)(23)	*uuuddduuuddd* 0.27152

a Three nonequivalent permutation types are identified. Labels 1–3 represent Co1, Co2, and Co3, while label 4 represents the Er4 site. The label 1234 simply refers to the Co1–Co2–Co3–Er4 site ordering.

**Figure 4:**
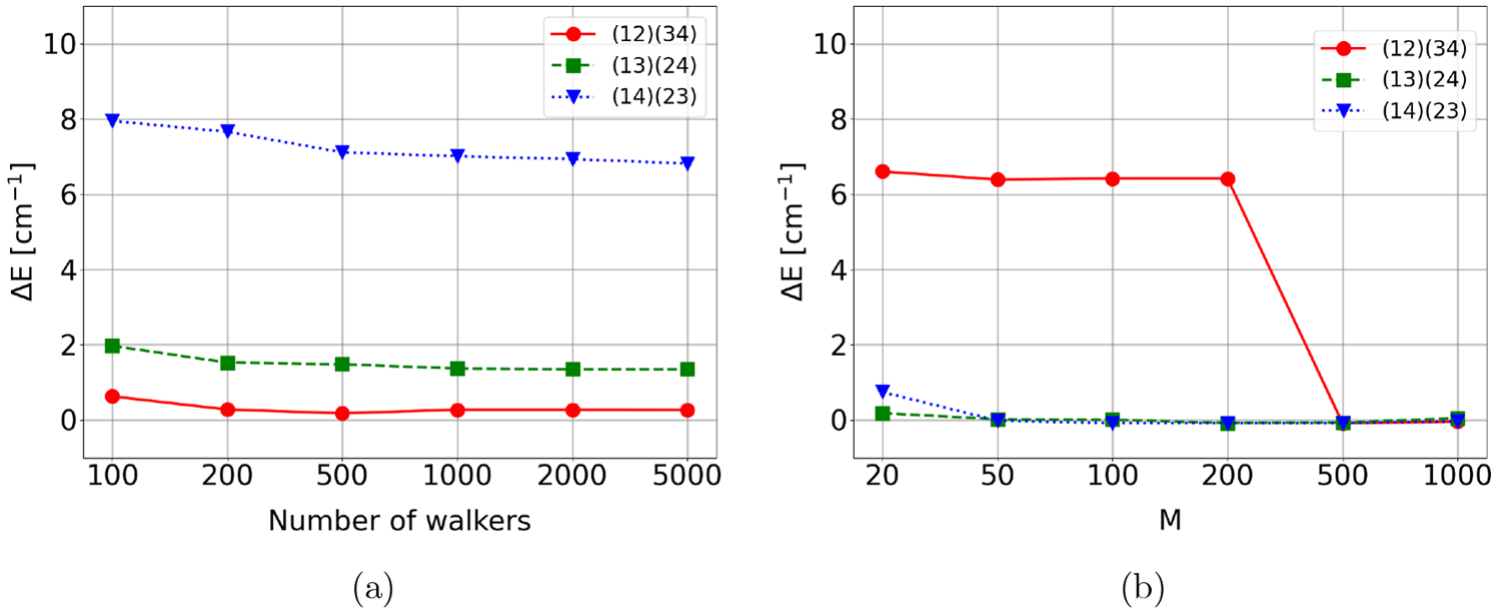
Deviations from the exact CAS(12,12) singlet energy using (a) FCIQMC(12,12) at increasing value of walker population (from 100 to 5000) and (b) DMRG(12,12) at increasing values of the bond dimension, *M*. In both cases, three ordering types, (12)(34), (13)(24), and (14)(23), are utilized.

The three site permutation types have further been studied within the FCIQMC and the DMRG frameworks. Figure 4a shows the energy convergence of FCIQMC(12,12) as a function of the number of walkers. In line with the conclusions drawn from Table 1 and Figure 3, the (12)(34) permutation type provides the fastest convergence with the number of walkers. The size of the CAS(12,12) is relatively small, containing only 226,512 CSFs, and while the (12)(34) ordering type converges already with 200 walkers, the (14)(23) exhibits a very slow convergence, and even 5,000 walkers are not sufficient to reach convergence. Figure 4b shows the convergence pattern of DMRG with respect to the site permutations. Interestingly, the (13)(24) and (14)(23) orderings converge faster than the (12)(34) permutation type with respect to *M*, confirming that the speed of convergence of GUGA-FCIQMC and DMRG with respect to the site reorderings is driven by different elements. An equivalent conclusion was obtained for one-dimensional spin systems.^60^

Thus, based on the analysis above the (12)(34) ordering is a good candidate as optimal ordering for the GUGA-FCIQMC optimization, while the other two ordering types are to be preferred within the DMRG framework.

Next, we turn our attention to the optimal ordering for the enlarged CAS(56,56). The ordering for the entire list of the 56 orbitals is summarized in Table 2.

**Table 2:** Orbital Orderings Utilized for FCIQMC(56,56) and DMRGCI(56,56) Calculations^a^

calculation	ordering
FCIQMC(56,56)	O9(2p×3)–O10(2p×3)–O11(2p×3)–O12(2p×3)–Co1(**3d**×3, 3d×2, d′×5)–Co2(d′×5, 3d×2, **3d**×3)–Co3(**3d**×3, 3d×2, d′×5)–Er4(f′×7, 4f×4, **4f**×3)
DMRGCI(56,56)	Co2(3dd′3dd′d′**3d**d′**3d**d′**3d**)–O11(2p×3)–Co3(d′3dd′**3d**3dd′**3d**d′**3d**d′)-O12(2p×3)–O10(2p×3)–Co1(d′**3d3d**d′d′3d**3d**d′3dd′)–O9(2p×3)–Er4(f′f′f′**4f**4f**4f**f′**4f**4f4ff′f′4ff′)

a The singly occupied orbitals are marked in bold.

For the singly occupied orbitals, the best ordering within the GUGA-FCIQMC algorithm reflects the ordering found for the CAS(12,12), namely (12)(34).

Noteworthy, within the GUGA-FCIQMC framework, the ligand orbitals are grouped together and separated from the metal centered orbitals, while the doubly occupied and empty orbitals of each metallic site precede and follow the singly occupied orbitals on the same site. This ordering reflects the one identified as optimal for the Fe_4_S_4_ cubane.^2^

Within the DMRG framework, algorithms have been advised for automating the process of finding the optimal site ordering. Examples are the Fiedler^83^ and the GA,^35^ also utilized in this work. Notably, these reordering algorithms themselves depend on the initial conditions, meaning that different starting orbital orderings might lead the Fiedler or GA algorithms to suggest different optimal orderings. Five different orderings of localized orbitals have been tested within the DMRG(56,56) *M* = 1000 calculations for *S*_tot_ = 0 spin state (see Table S5 for reference). We found that the optimal ordering for FCIQMC(56,56), followed by the GA gives the optimal orbital ordering for DMRG. This ordering is given in Table 2, and it is substantially different from the optimal ordering used for GUGA-FCIQMC.

Interestingly, the optimal ordering for the DMRG approach features the bridging 2p orbitals between the metal-ion orbitals and empty and doubly occupied orbitals admixed with the magnetic orbitals. Moreover, in the optimal orbital ordering for DMRG, the sequence of the singly occupied orbital of the metal ions corresponds to the (14)(23) type, which is the optimal DMRG orbital ordering for the smaller CAS(12,12).

Based on the optimal orbital ordering for FCIQMC, reported in Table 2, we optimized the CAS(56,56) wave functions for the *S*_tot_ = 0–6 spin states using FCIQMC.

In Table 3 we show the leading weights for each of the spin states, using both the CAS(12,12) and the CAS(56,56) active spaces.

**Table 3:** Dominant CSF(s) and Weight(s) Calculated from CASCI(12,12) and FCIQMC(56,56) for All Spin States Using the Co1–Co2–Co3–Er4 Ordering and the O9–O10–O11–O12–Co1–Co2–Co3–Er4 Ordering, Respectively^a^

spin	dominant CSF(s)	weight (12,12)	weight (56,56)
6	*uuuuuuuuuuuu*	1.00000	0.76321
5	*uuuuuuuuu**duu***	0.39856	0.29972
	*uuuuuuuuu**udu***	0.32609	0.24545
	*uuuuuuuuu**uud***	0.27174	0.20427
4	*uuuuuuuuu**ddu***	0.40610	0.30537
	*uuuuuuuuu**dud***	0.32489	0.24406
	*uuuuuuuuu**udd***	0.26581	0.19971
3	*uuuuuuuuuddd*	0.99830	0.74782
2	*uuuuuu**duu**ddd*	0.38611	0.31895
	*uuuuuu**udu**ddd*	0.28976	0.23922
	*uuuuuu**uud**ddd*	0.22531	0.18620
1	*uuuuuu**ddu**ddd*	0.38417	0.33400
	*uuuuuu**dud**ddd*	0.27448	0.23851
	*uuuuuu**udd**ddd*	0.20592	0.17915
0	*uuuuuudddddd*	0.92808	0.74790

a Only singly occupied 3d/4f orbitals are shown in the CSFs of FCIQMC(56,56) calculations. The spins indicated in bold correspond to the paths in the genealogical branching diagrams of Figure 5 that contribute to the spin-adapted wave function for each given targeted spin state.

In the case of *S*_tot_ = 0, 3, and 6, only one CSF dominates the CI expansion, with the spin up and spin down well separated in each site (collinear states). In the case of *S*_tot_ = 1, 2, 4, and 5 (non-collinear states), three leading CSFs are found, with the spins flipping on the same site (marked in red in Table 3). The multireference character of the intermediate *S*_tot_ = 1, 2, 4, and 5 spin states can be graphically represented via the genealogical branching diagrams,^88,89^ reported in Figure 5. All possible electronic configurations associated with the unpaired electrons are represented by paths branching through the genealogical branching diagram. This feature makes these diagrams extremely useful in describing the leading electronic configurations in systems mostly dominated by spin-exchange interactions. In Figure 5, only the significant paths leading to *S* = 5, 4, 2, and 1 are highlighted. For example, in Figure 5a, *S*_tot_ = 5, the spins on Co1, Co2, and Co3 are parallel aligned (black circles) and lead to the partial *S* = 9/2. The last three electrons belonging to Er4 site branch in three possible ways (in agreement with the configurations reported in Table 3) leading to the total *S* = 5 spin. Similar arguments apply to the branching diagrams corresponding to the other spin states.

**Figure 5:**
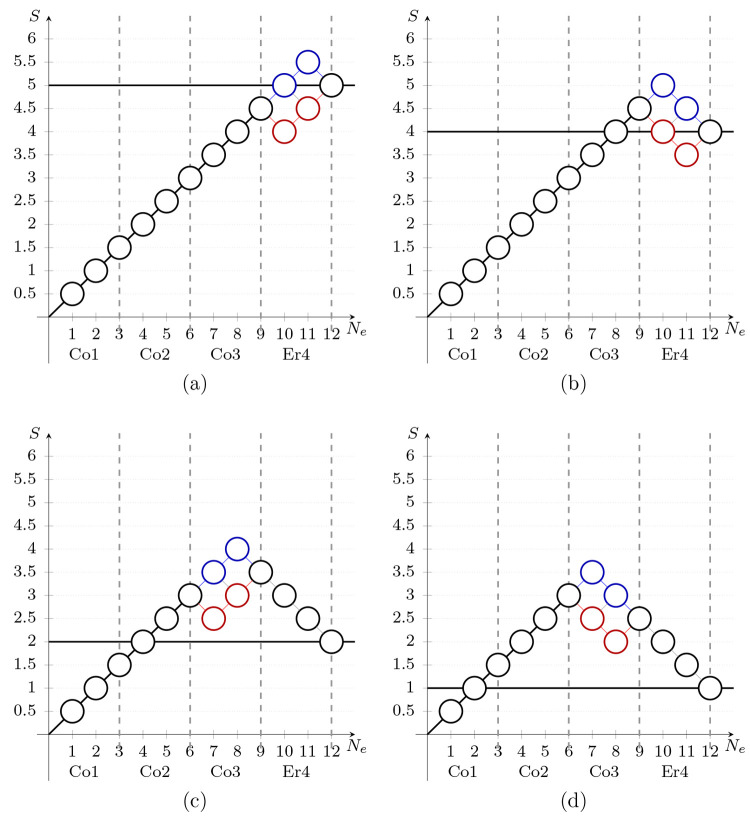
Genealogical branching diagrams describe the (a) *S*_tot_ = 5, (b) *S*_tot_ = 4, (c) *S*_tot_ = 2, and (d) *S*_tot_ = 1 spin states shown in Table 3. Each node represents a value of the total spin in the path, and blue and red nodes/lines shows the multireference character.

The strong multireference character of the *S*_tot_ = 1, 2, 4, 5 spin states is responsible for reduced convergence of GUGA-FCIQMC dynamics as a function of the number of walkers with respect to the predominant single-reference *S*_tot_ = 0, 3, and 6 spin states. An equivalent behavior has already been reported for some of the *S*_tot_ = 0 excited states of the Fe_4_S_4_ cubane models (see Figure 5 of ref (5)).

The weights of the leading CSFs for all spin states obtained at the CAS(12,12) and the CAS(56,56) level are rather similar. The general weight reduction for the CAS(56,56) wave function is to be related to the electron correlation outside the (12,12) active space, i.e., between doubly occupied and singly occupied 3d/4f orbitals, d′/f′ double-shell orbitals, and bridging oxygen 2p orbitals.

### CASSCF(12,12)//PT2(full) and CASSCF(12,12)//MCPDFT Spin Ladder

**Figure 6:**
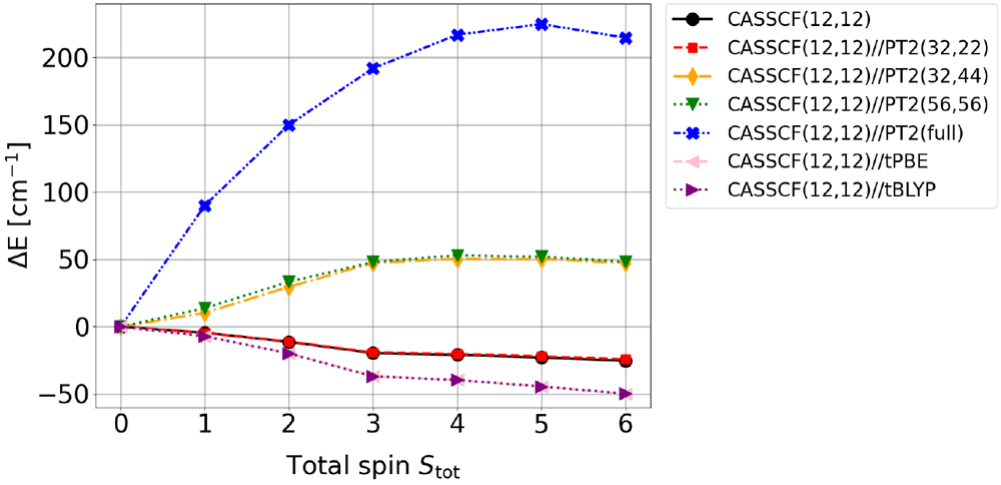
Spin gaps relative to the *S*_tot_ = 0 state at the CASSCF(12,12), CASSCF(12,12)//PT2(32,22), CASSCF(12,12)//PT2(32,44), CASSCF(12,12)//PT2(56,56), CASSCF(12,12)//PT2(full), CASSCF(12,12)//tPBE, and CASSCF(12,12)//tBLYP level.

The CASSCF(12,12)//PT2(full) and MCPDFT relative energies along the spin ladder are summarized in Figure 6 (see also Table S6). Noteworthy, the highest-to-lowest spin gaps predicted by CASPT2 and MCPDFT are very small (of the order of 200 cm^–1^), a clear manifestation of the challenges posed by the magnetic interactions of this study case. For energetically very close electronic states higher order electron correlation mechanisms might play a key role in differentially stabilizing one state over the others. This aspect has already been discussed in the context of correlation effects in an Fe(II)-porphyrin and a cuprate model system.^7,22^

Interestingly, CASSCF(12,12)//PT2(full) and MCPDFT predict qualitatively opposite relative stability of the *S*_tot_ = 0–6 electronic states, with the former suggesting an *S*_tot_ = 0 ground state and the latter (both with tPBE and tBLYP functional) an *S*_tot_ = 6 ground state.

The small deviation between the CASSCF(12,12) and the MCPDFT relative energies is to be related to fortuitous cancellation of errors in the former approach. In fact, the two methods are very different, with CASSCF accounting solely for correlation effects within the active space, while MCPDFT also accounts for correlation effects outside the active space.

As we will discuss in more detail in the next section, the discrepancy between CASPT2(full) and MCPDFT is to be attributed to the inability of PT2 in accounting for higher order correlation effects, such as oxygen-to-metal excitations and spin-coupling correlation effects across the excited unpaired electrons. Within the PT2 formalism, this limitation could in principle be reduced (but never eliminated) by enlarging the reference active space, thus including the higher order electron excitations explicitly into the CAS reference wave function. MCPDFT deals with these forms of correlation via the translated functionals and does not require an explicit treatment of higher order forms of correlation via excitation operators. As we will discuss below, larger active space calculations, based on DMRG and FCIQMC, numerically confirm the limitations of the CASPT2 approach and qualitatively support the results predicted by the MCPDFT procedure.

The 44 states of the low-energy spin manifold have also been investigated at the CASSCF(12,12)//PT2 and CASSCF(12,12)//MCPDFT levels.

Despite some changes in the relative stability of the excited states within the same spin multiplicity, our multistate CASPT2 calculations confirm the anti-ferromagnetic ordering for the model system investigated. In particular, the lowest *S* = 0 and the highest *S* = 6 states predicted by PT2 are the ones characterized in detail in Figure 8 and Figure 9 below. At the MCPDFT level, we observe a relative stability of the excited states that is not aligned with the ordering predicted by CASPT2. This result is not surprising, considering that already for the lowest spin ladder CASPT2 predicts anti-ferromagnetic ordering (erroneously, as discussed below), while MCPDFT predicts a ferromagnetic ordering of the low-energy states. The CASSCF(12,12)//tPBE results for the 44 low-energy states confirm the *S* = 6 ground state for this model system. The precise characterization of the entire set of 44 low-energy states goes beyond the scope of the present paper and will be presented in a separate publication together with the extraction of the magnetic coupling constants and some prediction of other magnetic properties, such as the temperature dependency of the magnetic susceptibility.

### CASSCF(12,12)//PT2(*X*,*Y*) Spin Ladder

The CASSCF(12,12)//PT2(*X*,*Y*) spin-gap predictions are summarized in Figure 6 (see also Table S7). The energetic trend of CASSCF(12,12)//PT2(32,22) is nearly indistinguishable from the CASSCF(12,12) spin ladder, suggesting that the electron correlation effects covered by PT2 within the same subshell (excitations from doubly to singly occupied valence 3d/4f orbitals) have a negligible differential contribution to the spin gap. Correlation effects introduced by the double-shell orbitals in the CASSCF(12,12)//PT2(32,44), however, have a strong differential effect, at least at the PT2 level of theory, and the relative stability of the low-energy spin-states reverses, revealing an *S*_tot_ = 0 ground state. The CASSCF(12,12)//PT2(56,56), in which also the valence 2p electrons of the bridging oxygen atoms are correlated at the PT2 level, shows a spin ladder that is very close to the smaller CASSCF(12,12)//PT2(32,44), indicating that the 2p orbitals from the bridging oxygens do not contribute differentially to the spin gap (very unlikely considering superexchange-like mechanisms) or that these forms of correlation are not well described at the PT2 level. Qualitatively, the CASSCF(12,12)//PT2(32,44) and the CASSCF(12,12)//PT2(56,56) reveal an energetic ordering comparable to the conventional CASSCF(12,12)//PT2(full).

Any limitation present in the CASSCF(12,12)//PT2(full) is already present in the smaller CASSCF(12,12)//PT2(56,56), allowing us to make a more direct comparison to DMRG and FCIQMC within the (56,56) active space, methods that are not limited by the electron excitation order, as opposed to the PT2 approach, and whose accuracy can be gradually increased, by enlarging the bond dimension value, *M*, and the number of walkers, respectively. These aspects will be discussed in greater detail in the following section.

### Large CASCI(56,56): Relative Stability of the *S*_tot_ = 0, 3, and 6 Spin States

**Figure 7:**
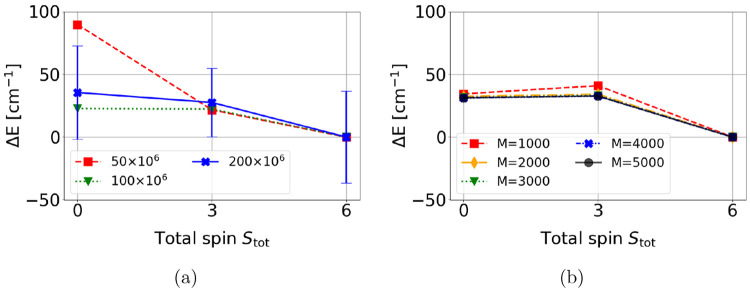
Relative energy of the *S*_tot_ = 0, 3, 6 spin states using (a) FCIQMC(56,56) and (b) DMRGCI(56,56) approaches. The standard deviation for the FCIQMC(56,56) dynamics using 200 × 10^6^ walkers is given as vertical bar for each spin state. The energies of *S*_tot_ = 6 spin state are set to 0.

In this section, we discuss the relative stability of three spin states, *S* = 0, 3, and 6, using the large CAS(56,56) active space and relying both on GUGA-FCIQMC (Figure 7a) and DMRG (Figure 7b).

The relative energy of the intermediate *S*_tot_ = 1, 2, 4, and 5 spin states has been omitted due to the suboptimal convergence, due to the inherently more multireference nature of these states (as shown in Table 3). GUGA-FCIQMC dynamics with 50 × 10^6^ walkers provide already a consistent trend in the calculated relative energies, with the high-spin state predicted as the ground state for this cubane system.

The DMRG approach (Figure 7b) also predicts a high spin *S*_tot_ = 6 ground state. In both methods, the highest-to-lowest spin gap amounts to a mere ∼35 cm^–1^. The only appreciable difference between the GUGA-FCIQMC and the DMRG predictions is that in the latter approach a monotonous energy trend is not observed, with the *S*_tot_ = 0 state being slightly more stable than the *S*_tot_ = 3 state. Differences are marginal, amounting to a few cm^–1^. However, the monotonous trend observed for the relative energies both at the MCPDFT (see Figure 6) and at the GUGA-FCIQMC(56,56) level might suggest that DMRG is not fully converged.

The CAS(56,56) relative energies obtained both at the FCIQMC and the DMRG level are to be compared to the CASSCF(12,12)//PT2(56,56). The orbital spaces are identical, yet the PT2(56,56) predicts an anti-ferromagnetic ordering, while FCIQMC and DMRG suggest a ferromagnetic ordering of the states. This result is a clear numerical demonstration of the limitation of PT2 approaches and that higher order correlation effects, which are not included at the PT2 level, can be crucial for qualitatively correct magnetic predictions. This error is amplified at the CASSCF(12,12)//PT2(full) level, which predicts an even stronger anti-ferromagnetic ordering of the low-energy states. Relying on the favorable scaling of MCPDFT with the size of the active space, as opposed to CASPT2, and considering that MCPDFT only relies on two-body density matrices, available both from GUGA-FCIQMC and DMRG, it has been possible to obtain CAS(56,56)/MCPDFT energies. We report the DMRG(56,56)//MCPDFT (*M* = 1000) total energies for *S*_tot_ = 0 and *S*_tot_ = 6 in Table 4, using both the tPBE and the tBLYP translated functionals. The method suggests a high-spin ground state with the highest-to-lowest energy gap of ∼44 cm^–1^. This spin gap prediction is in line with the CAS(56,56) results, using both FCIQMC and DMRG, and with the CAS(12,12)/MCPDFT approach. We note, however, that the results we discuss here are all ground-state calculations. Further studies may include more roots, investigating state swapping in more detail, and aim for a more precise spin ladder.

**Table 4:** Total and Relative Energies at the DMRGCI(56,56), DMRGCI(56,56)//tPBE, and DMRGCI(56,56)//tBLYP Level of Theory for *S*_tot_ = 0 and *S*_tot_ = 6^a^

spin state (*S*)	DMRGCI(56,56)	DMRGCI(56,56)//tPBE	DMRGCI(56,56)//tBLYP
6	–19449.810321	–19467.174788	–19470.859036
0	–19449.810159	–19467.174585	–19470.858834
Δ*E* [cm^–1^]	35.6	44.6	44.3

a M = 1000 is utilized for the preceding DMRG calculations. Total energy values are reported in hartree.

A [Co_3_Er] cubane with the same core structure and similar associated ligands has been experimentally studied and reported in the literature.^90^ The structure studied in the present work is not to be considered a close mimic of the experimental one. The experimental compound is 8-coordinate at the Er center, while the present study uses a 6-coordinate Er center. This change in coordination has been reported and discussed in previous DFT and DFT-based MD investigations (see refs (62 and 63)), where we have shown that an easy coordination change is energetically possible. The present work focuses on the 6-coordinate model compound extracted from refs (62 and 63). Despite the difference in coordination number at the Er center, also the experimental compound was reported as having a very weak ferromagnetic coupling, in line with the results reported here. Our results are quite promising and can be considered a unique computational prediction on a 6-coordinate [Co_3_Er] cubane with possible extensions to other comparable cubane systems.

Moreover, synthetic and model [Mn(IV)_3_CaO_4_] cubanes (mimics of the photosystem II active site) have also shown preference for high spin ground states.^13^

### Local Spin and Spin–Spin Correlation

Local spin and spin–spin correlation expectation values are calculated for spin states *S*_tot_ = 0–6 on the basis of the FCIQMC(56,56) wave functions. The local spin operator of orbital *i*, *Ŝ*_*i*_^2^, and the spin–spin correlation operator between orbitals *i* and *j*, *Ŝ*_*i*_·*Ŝ*_*j*_, in the spin-free formulation are defined as^2^
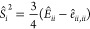
2and
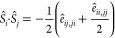
3respectively, where *Ê*_*ij*_ and *ê*_*ij*,*kl*_ are the spin-free one- and two-electron excitation operators.^72^ Their expectation values, ⟨*Ŝ*_*i*_^2^⟩ and ⟨*Ŝ*_*i*_·*Ŝ*_*j*_⟩, can be obtained in a contracted form directly from the one- and two-body reduced density matrices. The local spin of a magnetic center , , and the spin–spin correlation between magnetic centers  and , , can be obtained by combining eqs 2 and 3 as
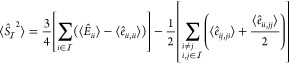
4and
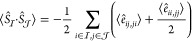
5where  and  in general represent the sets of localized orbitals from two sites (e.g., atoms, ions, etc.) fulfilling the condition . The algebraic method to derive eqs 2–5 has been discussed in detail in Appendices C and D of ref (2).

The spin–spin correlation expectation value can also be calculated using the local spin expectation values of two sites as
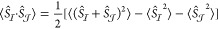
6

**Figure 8:**
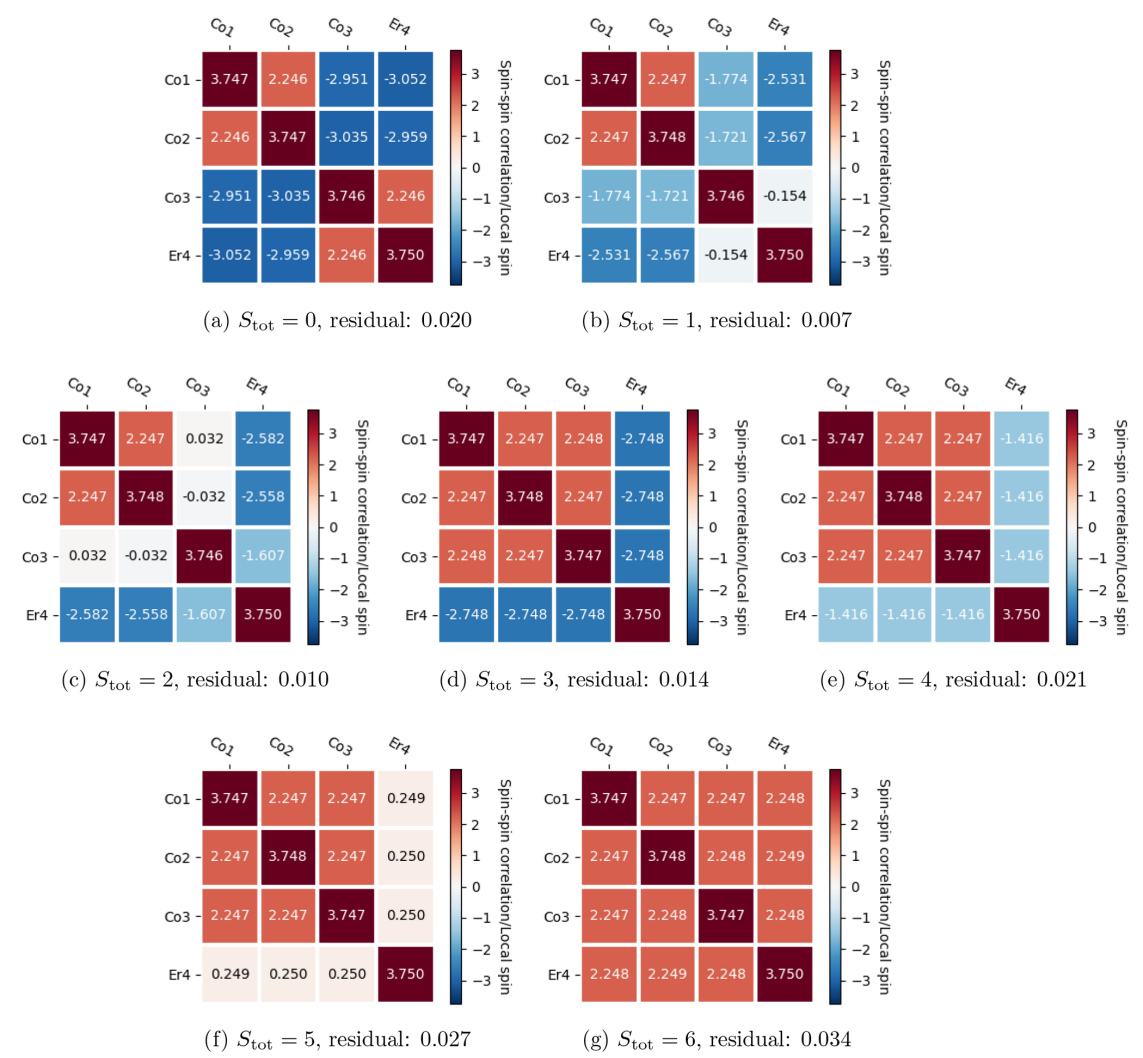
Local spin of metal centers and spin–spin correlation between metal centers in the FCIQMC(56,56) calculations for *S*_tot_ = 0–6, respectively. The diagonal part of each panel provides the local spin of each site and the off-diagonal part provides the spin–spin correlation between sites. The residual value reported for each spin state shows the difference between the formal spin expectation values (*S*(*S* + 1)) and the cumulative spin sum on the four metal sites. This value indicates the role of the bridging oxygens in delocalizing the spin.

Figure 8 shows the local spin and the spin–spin correlation expectation values for the four metal centers in the basis of the FCIQMC(56,56) wave functions for the *S*_tot_ = 0–6 spin states and using 200 × 10^6^ walkers. The diagonal part of each panel provides the local spin of each site , and the off-diagonal part provides the spin–spin correlation between sites  (see also Figure S1).

The sum of 16 numbers in each panel approaches the total spin expectation value *S*(*S* + 1) for the seven *S*_tot_ = 0–6 spin states investigated. The deviations from the corresponding formal *S*(*S* + 1) values are to be attributed to spin fluctuations involving the four bridging oxygen atoms. These residual values are reported for each spin state in Figure 8. The residual values change across the spin states but in general remain small, indicating a relatively small delocalization of spin on the bridging atoms. We note that the local spin expectation value for each metal center approaches the formal 3/2 × (3/2 + 1) = 3.75 value. This result indicates negligible ligand-to-metal charge-transfer (MLCT) and weak ligand field effects, that make contributions from non-Hund electronic configurations to the wave function vanishingly small.

The individual values within the blue cells in Figure 8 do not represent the direct spin–spin correlation between two pure local 3/2-spins, as in the case of the values within the red blocks. We explain this observation with an example. Let us consider four *S*_loc_ = 1/2 sites, A, B, C, and D, with A and B parallel aligned, *S*_AB_ = 1, C and D parallel aligned, *S*_CD_ = 1, and the two AB and CD pairs antiparallel aligned (*S*_ABCD_ = 0). The 2 × 2 diagonal blocks consist of local spin with values 0.75 (diagonal entries) and spin–spin correlation values ⟨*Ŝ*_A_·*Ŝ*_B_⟩ = ⟨*Ŝ*_C_·*Ŝ*_D_⟩ = 0.25. If the eight remaining off-diagonal elements referred to the spin–spin correlation values between pure doublet states, they would have had a value of 1/2 × (0 – 1/2(1/2 + 1) × 2) = −0.75 (relying on eq 6). The sum of the 16 elements should equal the total spin expectation value and, as per the above assumption, this sum equals 0.75 × 4 + 0.25 × 4 – 0.75 × 8 = −2, which is in obvious contradiction with the correct expectation value for the total singlet, *S*(*S* + 1) = 0.

Nonetheless, the partial sums (column-wise or row-wise) of the off-diagonal elements lead to important physical quantities. Similarly to eq 6, we can write the following equations for two, three, and four sites:
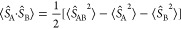
7
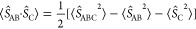
8
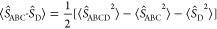
9Elements ⟨1·2⟩, (⟨1·3⟩ + ⟨2·3⟩), and (⟨1·4⟩ + ⟨2·4⟩ + ⟨3·4⟩) can be related to eq 7, eq 8, and eq 9, respectively. Thus, the column-wise (or row-wise) sums relate to the cumulative ⟨*Ŝ*_A_·*Ŝ*_B_⟩, ⟨*Ŝ*_AB_·*Ŝ*_C_⟩, and ⟨*Ŝ*_ABC_·*Ŝ*_D_⟩ spin–spin correlation values. To obtain the individual value of, e.g., ⟨*Ŝ*_A_·*Ŝ*_C_⟩ (or ⟨*Ŝ*_Co1_·*Ŝ*_Co3_⟩ in Figure 8a), one needs to expand the wave function in Slater determinants (see Appendix below).

In the following paragraphs, we discuss the spin–spin correlation values for each individual spin state in more detail.

#### ***S*_tot_ = 0**

Figure 8a exhibits a ⟨*S*_Co1_·*S*_Co2_⟩ (and ⟨*S*_Co3_·*S*_Er4_⟩) spin–spin correlation value in very close agreement to the formal value that would be obtained from eq 6 for two parallel aligned quartet local spins, namely, ⟨*S*_Co1_·*S*_Co2_⟩ = 2.25. The sum of the four (red) entries in the Co1–Co2 2 × 2 upper-left diagonal block, as well as the sum of the four (red) entries in the Co3–Er4 2 × 2 lower-right diagonal block in Figure 8a, both approaching the value ∼12, also suggest a formal system of two parallel aligned quartet spins, *S*_pair_ = (3/2 + 3/2) = 3, whose total spin expectation value is 3(3 + 1) = 12. The sum of the four entries of each 2 × 2 off-diagonal block (blue upper-right and lower-left blocks in Figure 8a) approaches the −12 value, which matches (from eq 6) the formal value of , with  and  pairs, exhibiting spin parallel alignment within the pairs, thus , and a global antiparallel alignment. The spin interactions within the cubane in *S*_tot_ = 0 state can then be treated as the antiparallel alignment between two spin vectors *S*_Co1Co2_ = 3 and *S*_Co3Er4_ = 3. The local spin and the spin–spin correlation values confirm the physical interpretation that we extracted from the corresponding highly compressed and mostly single-reference wave function (see Table 3).

#### ***S*_tot_ = 1 and *S*_tot_ = 2**

Figure 8b,c show the ferromagnetic spin alignment between Co1 and Co2, with a 2 × 2 upper-left block very similar to the corresponding block for the *S*_tot_ = 0 spin state. However, the remaining of the two matrices are substantially different from the *S*_tot_ = 0 case discussed above. The sum of the first two elements of the third column and the sum of the first three elements of the last column in Figure 8b are in very good agreement with the formal values obtained from eq 8 (⟨*S*_AB_·*S*_C_⟩ = −3.5) and eq 9 (⟨*S*_ABC_·*S*_D_⟩ = −5.25), respectively, considering *S*_ABCD_ = 1, *S*_ABC_ = 5/2, ⟨*S*_A_·*S*_B_⟩ = 9/4, and *S*_A_ = *S*_B_ = *S*_C_ = *S*_D_ = 3/2. Similar considerations apply to Figure 8c.

#### ***S*_tot_ = 3–6**

Figure 8d–g is consistent with the ferromagnetic spin alignment between Co1, Co2, and Co3, featuring maximal spin–spin correlation expectation values ∼2.25 for each entry. The sum of the nine elements of the 3 × 3 upper-left block indicates parallel spin alignment across the first 3 sites (*S*_ABC_ = 9/2). With the increase of the total spin from *S*_tot_ = 3 to *S*_tot_ = 6, the spin–spin correlation between Co1–Co2–Co3 and Er increases monotonously, as expected from eq 9.

**Figure 9:**
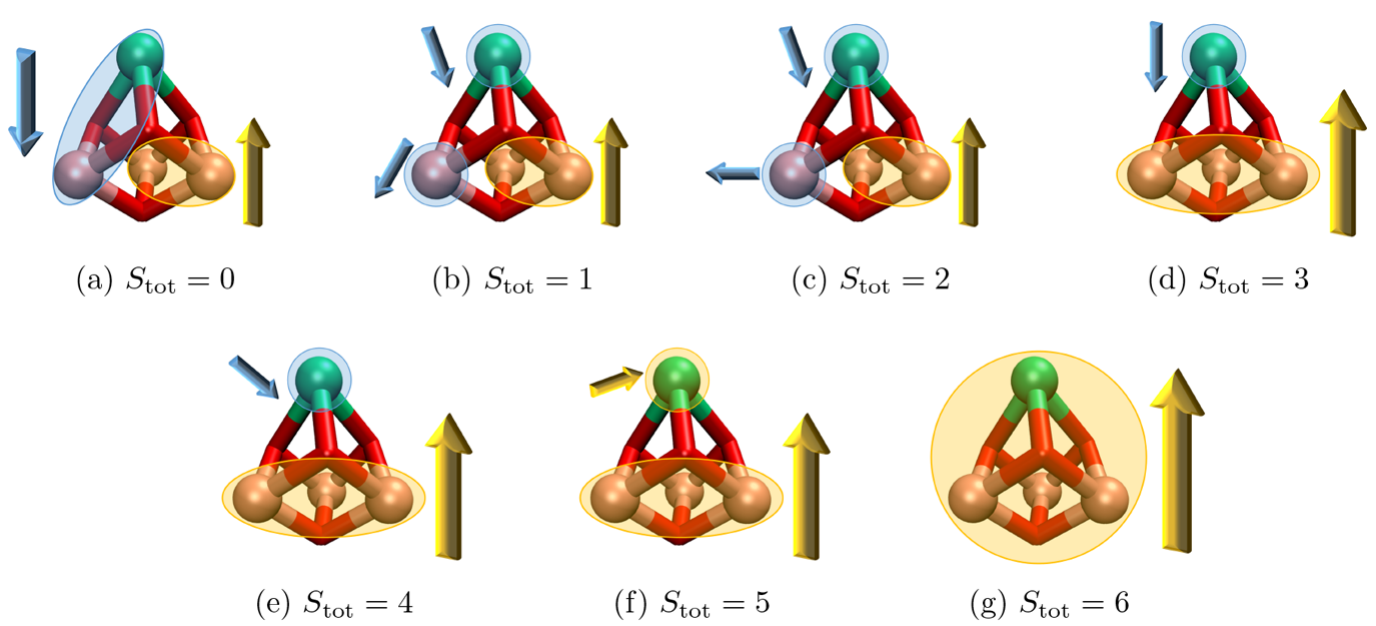
Illustration of the spin interactions across the four magnetic sites from spin state (a) *S*_tot_ = 0 to (g) *S*_tot_ = 6. Pink: cobalt, green: erbium, red: oxygen. The arrow represents the cumulative spin vector on one or multiple magnetic centers.

The analysis of the multiconfigurational wave functions, summarized in Table 3, and the spin–spin correlation values reported in Figure 8 clearly indicate that in climbing the spin ladder from *S*_tot_ = 0 to *S*_tot_ = 6 in the investigated [Co_3_Er(OR)_4_] cubane model (a) a strong ferromagnetic interaction between Co1 and Co2 exists that is not altered along the spin ladder, (b) the spin at the Co3 site gradually aligns (parallel) with the Co1–Co2 pair as the total spin increases, (c) for *S*_tot_ = 3 the spins at Co1, Co2, and Co3 are perfectly aligned (*S*_Co1Co2Co3_ = 9/2) while the spin at the Er4 site is antiferromagnetically oriented to the former sites, and (d) for *S*_tot_ = 4–6 the spin at the Er4 site gradually aligns to the Co1–Co2–Co3 spins while the spins at the Co1–Co2–Co3 sites remain perfectly parallel aligned. A more qualitative illustration of the spin flipping along the spin ladder is shown in Figure 9.

Notably, the spin interactions along the spin ladder for the cubane here investigated are different from the ones in the [Fe_4_S_4_] cubane studied in an earlier work.^2^ While in the [Co_3_Er(OR)_4_] cubane in the high spin sectors (*S* = 3–6) the three Co sites are parallel aligned and the Er4 site is responsible for the spin changes, in the [Fe_4_S_4_] cubane the interaction between the two pair vectors (AB and CD sites) has a stronger role, and as opposed to the asymmetricity found for Figure 9, the two spins in AB and CD sites couple their *rigid* spins, spanning the entire spin ladder. This different behavior becomes obvious when comparing the ⟨(*S*_A_ + *S*_B_ + *S*_C_)^2^⟩ total spin of Figure 8 with the corresponding values from Figure 15c of ref (2).

The local spin and the spin–spin correlation analysis represent good guidelines for understanding purely from first-principles magnetic interactions in polynuclear transition metal clusters.

## Conclusions

In this work, a protocol has been presented, based on forefront multireference methodologies especially designed for systems featuring strong electron correlation effects, for investigating the magnetic properties of a [Co(II)_3_Er(III)(OR)_4_] cubane model. Seven of the lowest-energy electronic states, with spin in the range *S*_tot_ = 0–6 have been investigated using conventional CASSCF(12,12), as well as large active space eigensolvers such as DMRG and FCIQMC. Active spaces as large as CAS(56,56) have been utilized for understanding the role of various forms of electron correlation. Methods specifically designed for dynamic correlation effects, on top of multireference wave functions have also been employed, namely, the conventional CASSCF(12,12)/CASPT2 (only on top of small active space reference wave functions) and MCPDFT (for both small and larger active space reference wave functions). With these methods at hand the relative stability of the seven spin states has been studied.

At the small CAS(12,12) level, we observe important discrepancies between CASPT2 and MCPDFT. CASPT2(12,12) suggests a low-spin ground state, an anti-ferromagnet; MCPDFT(12,12) predicts a high-spin ground state, a ferromagnet.

At the CAS(56,56) level, both DMRG and FCIQMC algorithms suggest a very small highest-to-lowest spin gap (∼35 cm^–1^), and both predict a high-spin ground-state (*S*_tot_ = 6). Complementing the large CAS(56,56) with the MCPDFT(56,56) does not affect appreciably the highest-to-lowest spin gap (∼44 cm^–1^) as compared to DMRG and FCIQMC predictions, albeit there is a further differential stabilization of the high-spin state. We note that our procedure was carried out at fixed CAS(12,12) variational optimized orbitals and at a unique geometry. Factors that could influence the theoretical prediction and that have not been considered in the present work include orbital relaxation effect at the larger CAS(56,56) active space, spin–orbit coupling, geometry optimization for different spin states, and last but not least environmental effects and finite temperature. Co-3d^7^ electronic configurations in an octahedral environment generally feature a ^4^*T*_1_ ground state, and possess a first-order orbital moment **L**. Hence, spin–orbit coupling effects are important, and in principle should not be neglected when describing the magnetic properties involving such magnetic centers. For quasi-octahedral environments, such as the one characterizing our model cubane, the degeneracy of the three components of the *T*_1_ state might in part be lifted, but depending on the degree of distortion the spin–orbit coupling might still play a significant role. Similar effects characterize the Er-4f^11^ configuration. Adding these contributions to the CAS(56,56) reference wave function, however, adds significantly to the computational efforts, and it is currently practically unattainable. Additionally, spin–orbit coupling effects are currently out of reach for the large active space reference wave functions. Work is in progress in this direction and will be discussed in a future work. Already the solution of the CAS(56,56) CI problem required special theoretical efforts to make the DMRG and FCIQMC algorithm approach convergence relatively quickly. Notably, orbital localizations and reorderings have been deemed necessary for both algorithms.

We found that the best orbital orderings for FCIQMC and DMRG differ. This aspect is in line with earlier observations for simpler one-dimensional systems (chain of hydrogens).

In order to further characterize the magnetic interactions within the [Co(II)_3_Er(III)(OR)_4_] model cubane, local-spin and spin–spin correlation values have been analyzed in great detail.

Starting from the highest spin state (*S*_tot_ = 6), as lower total spin states are considered, first the Er spin is forced into the anti-ferromagnetic alignment, and only for spin states with *S* < 3 also the Co3 spin is forced to change direction with respect to the Co1–Co2 pair. These spin interactions are very different from the ones observed in the more symmetric Fe_4_S_4_ cubane,^2,5^ which is better described as the interaction of two Fe_A_–Fe_B_ and Fe_C_–Fe_D_ pairs, with quasi-rigid parallel spin alignment within the pair. A ferromagnetic ground state is in line with a previous experimental study of a structurally similar [Co_3_Er] cubane.^90^

The multiconfigurational strategy here presented, with emphasis on the importance of orbital localization and orderings, represents a novel theoretical tool to predict, at least qualitatively, the relative stability of quasi-degenerate electronic states and spin interactions between the magnetic centers, providing useful insights into the magnetic properties of these systems. Moreover, the resulting energetically close-lying spin states of [Co(II)_3_Er(III)(OR)_4_] are advantageous for efficient catalysis and are in line with previous findings that the highly complex polynuclear transition metal cubane complex is one of the rare bioinspired active and stable cubane water oxidation catalysts.

## Appendix

We can express the spin configuration of A and B sites as Slater determinants:

10

where α and β represent the *m*_s_ = +1/2 and *m*_s_ = −1/2 spin projections for single electrons, respectively. Three expressions representing different *S*_AB_^*z*^ contribute to the same spin configuration.

Similarly, *S*_ABC_ = 1/2 leads to six expressions in Slater determinants considering the permutations of A, B, and C sites: |α_A_α_B_β_C_⟩, |α_A_β_B_α_C_⟩, |β_A_α_B_α_C_⟩, |β_A_β_B_α_C_⟩, |β_A_α_B_β_C_⟩, and |α_A_β_B_β_C_⟩. However, the constraint of *S*_AB_ in eq 10 limits it to four expressions:

11

The wave function Ψ_ABC_ is the combination of the above four expressions, and we can consider the operator *Ŝ*_B_·*Ŝ*_C_ on them separately.

The spin–spin correlation of |α_A_α_B_β_C_⟩ is

12

|β_A_β_B_α_C_⟩ also takes −0.75 by symmetry.

The spin–spin correlation of  is

13

 also takes −0.25 by symmetry.

If the four expressions in eq 11 have the same weights in the wave function, ⟨*Ŝ*_B_·*Ŝ*_C_⟩ gives 1/4 × [2 × (−0.75) + 2 × (−0.25)) = −0.5. Similarly, ⟨*Ŝ*_Co1_·*Ŝ*_Co3_⟩ = ⟨*Ŝ*_Co2_·*Ŝ*_Co3_⟩ = −3 in the ideal (equally weighted) case, which are close to the values shown in Figure 8a.

## Supporting Information Available

Cartesian coordinates of [Co_3_Er(OR)_4_] cubane structure; CASSCF(12,12) optimization procedure for spin states *S*_tot_ = 0–6; Table S1, CASSCF(12,12) total energies for *S*_tot_ = 0–6 lowest electronic states; Table S2, orbitals kept frozen at the CASSCF(12,12)//PT2(full) level; Table S3, CASSCF(12,12)//PT2(full) energies for different FNO thresholds; Table S4, CASSCF(12,12)//PT2(full) energies for different IPEA shift values; Table S5, orbital ordering effect on DMRG convergence; Table S6, CASSCF(12,12)//PT2(full), CASSCF(12,12)//tPBE, and CASSCF(12,12)//tBLYP total energies for *S*_tot_ = 0–6; Table S7, CASSCF(12,12)//PT2(32,22), CASCI(12,12)//PT2(32,44), CASCI(12,12)//PT2(56,56) total energies for *S*_tot_ = 0–6; Figure S1, local spin and spin–spin correlation values; DMRG(56,56) input examples; GUGA-FCIQMC(56,56) input examples (PDF)
