# Supplementary material for: Magnetic Interactions in a [Co(II)3Er(III)(OR)4] Model Cubane through Forefront Multiconfigurational Methods
Source: J Chem Theory Comput. 2023 May 1;19(10):2811–26. doi: 10.1021/acs.jctc.2c01318 (PMC10210248; doi:10.1021/acs.jctc.2c01318)
Supplement: Supplementary file 1 — ct2c01318_si_001.pdf [file ct2c01318_si_001.pdf]

Supporting Information: Magnetic Interactions in a $[Co(II)_3Er(III)(OR)_4]$ Model Cubane Through Forefront Multiconfigurational Methods: Supporting Material

Ruocheng Han[†], Sandra Luber[*,†] and Giovanni Li Manni[*,‡]

†*Department of Chemistry A, University of Zurich, Winterthurerstrasse 190, 8057 Zurich, Switzerland*

‡*Max Planck Institute for Solid State Research, Heisenbergstrasse 1, 70569 Stuttgart, Germany*

E-mail: sandra.luber@chem.uzh.ch; g.limanni@fkf.mpg.de

S1 Geometry information

Cartesian coordinates. Unit: Å

Co 9.67500 9.23300 7.45300

Co 9.58100 7.13900 9.42200

Co 9.11500 10.05700 10.55900

Er 6.49700 8.88900 8.84400

N 10.37400 11.11300 7.11100

N 8.71100 9.05900 5.59000

N 10.68900 11.37700 10.56600

N 9.59800 9.25500 12.44800

O 8.26900 10.41400 8.61500

O 8.30300 7.78700 7.84000

O 10.49300 9.06500 9.36400

O 8.07200 8.30600 10.49000

O 11.28800 8.10800 6.73400

O 10.74000 6.25900 7.93300

O 7.71400 11.39100 11.32700

O 5.87100 10.39600 10.46400

O 5.85600 6.82800 9.23400

O 5.62900 9.49800 7.07100

O 10.52000 6.64400 10.95100

C 9.62000 12.12500 7.60300

C 10.10900 13.43000 7.64000

H 9.49100 14.22300 8.06300

C 11.39200 13.69900 7.15900

H 11.79400 14.71200 7.19300

C 12.15900 12.64600 6.65000

H 13.16800 12.81000 6.27200

C 11.61100 11.36800 6.64200

H 12.14900 10.49400 6.26800

C 8.24800 11.72000 8.10800

H 7.91700 12.44500 8.87700

H 7.52800 11.77700 7.26500

C 8.74400 9.92000 4.55900

H 9.34900 10.81500 4.71400

C 8.05900 9.69900 3.36900

H 8.11500 10.43000 2.56200

C 7.30200 8.53000 3.24200

H 6.74700 8.32300 2.32600

C 7.26100 7.63800 4.31200

H 6.67000 6.72200 4.26300

C 7.97500 7.93300 5.47400

C 8.00100 7.03100 6.68600

H 7.02800 6.52200 6.80500

H 8.78100 6.25900 6.54400

C 10.63600 12.65200 10.99900

H 9.65700 12.97100 11.36200

C 11.74500 13.49000 10.97900

H 11.66000 14.51700 11.33300

C 12.95500 12.98600 10.49000

H 13.84300 13.61800 10.44700

C 13.00800 11.66300 10.05000

H 13.93100 11.23800 9.65400

C 11.85800 10.87300 10.10600

C 11.80900 9.41700 9.68400

H 12.48500 9.25100 8.82400

H 12.16700 8.78300 10.52100

C 10.58800 9.62700 13.27600

H 11.16300 10.50100 12.96300

C 10.87500 8.95400 14.45700

H 11.69600 9.28700 15.09200

C 10.09100 7.84700 14.79900

H 10.28900 7.28900 15.71500

C 9.06600 7.45900 13.94200

H 8.44400 6.59100 14.16100

C 8.85300 8.17600 12.76200

C 7.76600 7.81800 11.77500

H 7.62900 6.72500 11.74700

H 6.81400 8.26600 12.12400

C 11.45300 6.89800 7.09500

C 12.58100 6.13100 6.42200

H 12.21700 5.75300 5.45500

H 12.89300 5.27700 7.03400

H 13.43000 6.79600 6.22100

C 6.46100 11.32000 11.10600

C 5.61300 12.44600 11.67200

H 5.98700 13.40700 11.29100

H 5.71900 12.46900 12.76600

H 4.55900 12.32200 11.40200

H 5.08600 6.30500 8.97100

H 5.33200 9.60300 6.16000

H 10.22200 5.76600 11.25100

O 8.08900 5.48100 9.58900

H 8.22700 4.89400 8.82700

H 7.17500 5.94300 9.43100

CASSCF(12,12) optimization procedure for spin states $S_{\text{tot}} = 0 - 6$

In Table S1, we provide total CASSCF(12,12) energies for spin states with $S_{\text{tot}} = 0 - 6$, starting from (1) the natural orbitals of the $S_{\text{tot}} = 6$ state (Natural), (2) the natural orbitals of the immediately higher spin state (Natural/Stepwise), and (3) the localized orbitals of the immediately higher spin state (Localized/Stepwise). Differences up to $\sim 50\mu$H are observed for the states of lower spin multiplicity ($S_{\text{tot}} = 0 - 1$). While small, these differences are large enough with respect to the highest-to-lowest spin gap at the CASSCF(12,12) level of theory. The effect of the initial conditions for spin state optimizations already for the smallest active space should raise a warning flag for the energetics obtained for larger active spaces. For this reason we deem the Localization and the stepwise optimization the procedure an important technical detail that should not be underestimated when calculating spin gaps in PNTM clusters.

Table S1: CASSCF(12,12) total energies for $S_{\text{tot}} = 0 - 6$ lowest electronic states, using (1) the natural orbitals of the highest spin state $S_{\text{tot}} = 6$ (Natural), (2) the natural orbitals of the immediately higher spin state (Natural/Stepwise), and (3) the localized orbitals of the immediately higher spin state (Localized/Stepwise). Unit: Hartree

Spin state (S)	Natural	Natural/Stepwise	Localized/Stepwise
6	-19449.387726	-19449.387726	-19449.387726
5	-19449.387715	-19449.387715	-19449.387715
4	-19449.387707	-19449.387706	-19449.387706
3	-19449.387700	-19449.387698	-19449.387700
2	-19449.387657	-19449.387661	-19449.387662
1	-19449.387586	-19449.387627	-19449.387631
0	-19449.387611	-19449.387580	-19449.387611

S2 Figures and Tables

Table S2: Orbitals Kept Frozen in the CASSCF(12,12)//PT2(full) calculations.

Atomic type	Inner shells
Er	1s2s2p3s3p3d4s4p
Co	1s2s2p
O	1s
N	1s
C	1s

Table S3: CASSCF(12,12)//PT2(full) total energies for different FNO thresholds. Spin $S_{\text{tot}} = 0$ and $S_{\text{tot}} = 6$ are tested and the energy difference between them is reported. Unit: Hartree

Threshold	E($S_{\text{tot}} = 0$)	E($S_{\text{tot}} = 6$)	E($S_{\text{tot}} = 6$) - E($S_{\text{tot}} = 0$)
0.6	-19458.137243	-19458.135674	0.001569
0.7	-19458.508793	-19458.507815	0.000978
0.8	-19458.786154	-19458.785167	0.000987
0.9	-19458.966580	-19458.965591	0.000989
1.0	-19459.034770	-19459.033784	0.000985

Table S4: CASSCF(12,12)//PT2(full) total energies for different IPEA shift values. Spin $S_{\text{tot}} = 0$ and $S_{\text{tot}} = 6$ are tested and the energy difference between them is reported. Unit of IPEA shift: Hartree, unit of energy: Hartree

IPEA shift	E($S_{\text{tot}} = 0$)	E($S_{\text{tot}} = 6$)	E($S_{\text{tot}} = 6$) - E($S_{\text{tot}} = 0$)
0.10	-19458.529019	-19458.528696	0.000323
0.25	-19458.508793	-19458.507815	0.000978
0.40	-19458.489585	-19458.488056	0.001529

Table S5: DMRGCI(56,56) M=1000 total energies for the $S_{\text{tot}} = 0$ state using different orderings of starting orbitals. *Non-reordered* means original orbital ordering (i.e. directly after construction of the active space or localization). *Reordered* means reordered as O9-O10-O11-O12-Co1-Co2-Co3-Er4 (see FCIQMC(56,56) ordering in Table 2 for more details). GA ordering represents the ordering based on the genetic algorithm, and Fiedler ordering represents the ordering based on the Fiedler vector.

Conditions	Energy [Hartree]
non-localized & non-reordered	-19449.52792294
non-localized & non-reordered + GA ordering	-19449.80886874
localized & non-reordered	-19449.69507447
localized & non-reordered + GA ordering	-19449.72224472
localized & reordered	-19449.80961538
localized & reordered + GA ordering	-19449.81015878
localized & Fiedler ordering	-19449.74797403

Table S6: CASSCF(12,12)//PT2(full), CASSCF(12,12)//tPBE, and CASSCF(12,12)//tBLYP Electronic Energies for $S_{\text{tot}} = 0 - 6$. Unit: Hartree

Spin state (S)	CASSCF(12,12)//PT2(full)	CASSCF(12,12)//tPBE	CASSCF(12,12)//tBLYP
6	-19458.507815	-19467.379312	-19471.064602
5	-19458.507769	-19467.379288	-19471.064578
4	-19458.507805	-19467.379265	-19471.064556
3	-19458.507919	-19467.379252	-19471.064544
2	-19458.508110	-19467.379175	-19471.064466
1	-19458.508383	-19467.379117	-19471.064408
0	-19458.508793	-19467.379085	-19471.064376

Table S7: CASSCF(12,12)//PT2(32,22), CASSCF(12,12)//PT2(32,44), CASSCF(12,12)//PT2(56,56) Electronic Energies for $S_{\text{tot}} = 0 - 6$. Unit: Hartree

Spin state (S)	CASSCF(12,12)//PT2(32,22)	CASSCF(12,12)//PT2(32,44)	CASSCF(12,12)//PT2(56,56)
6	-19449.388368	-19449.832742	-19449.844985
5	-19449.388358	-19449.832729	-19449.844968
4	-19449.388350	-19449.832728	-19449.844963
3	-19449.388345	-19449.832742	-19449.844986
2	-19449.388308	-19449.832823	-19449.845053
1	-19449.388279	-19449.832911	-19449.845142
0	-19449.388258	-19449.832958	-19449.845205

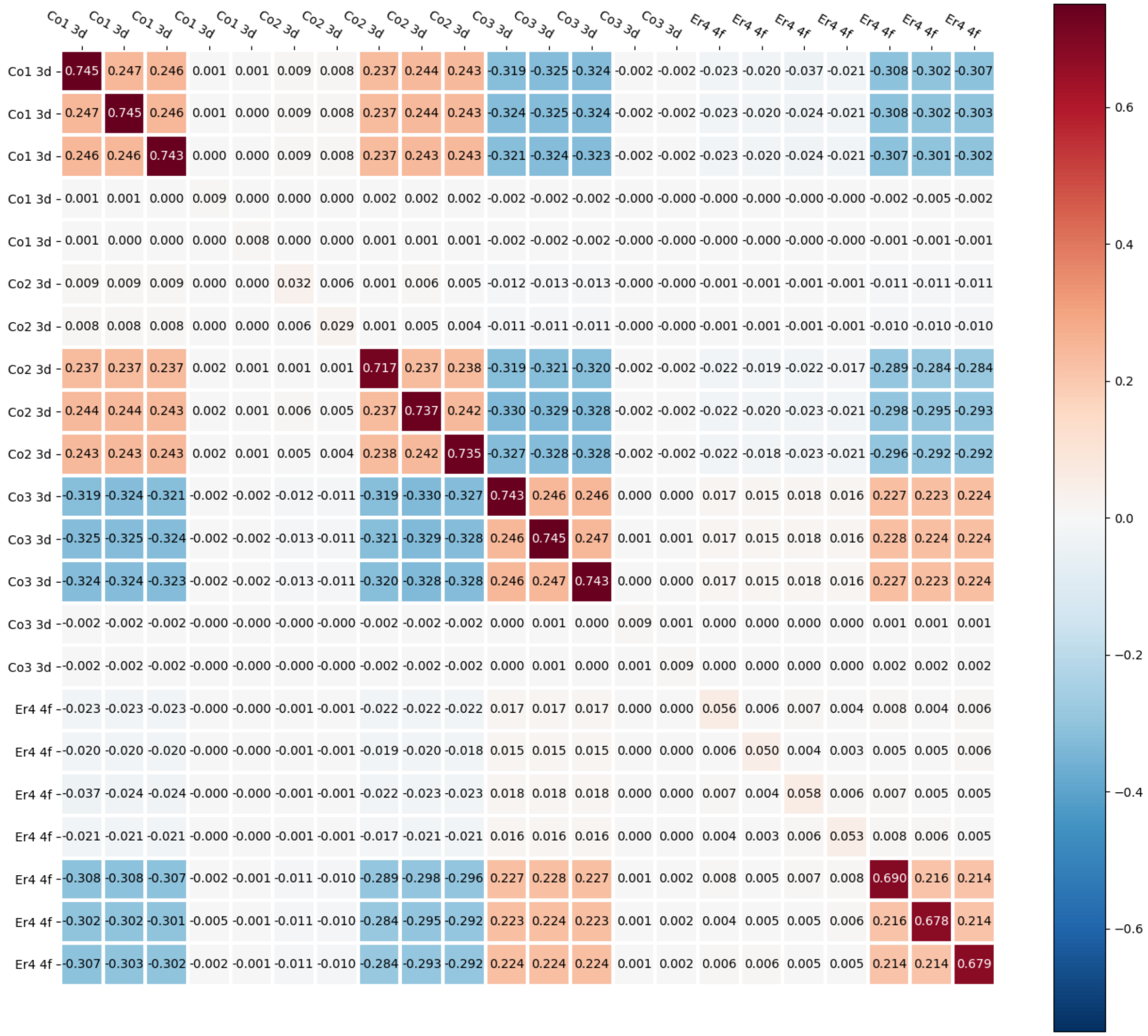

(a)

Figure S1: See next page for the caption

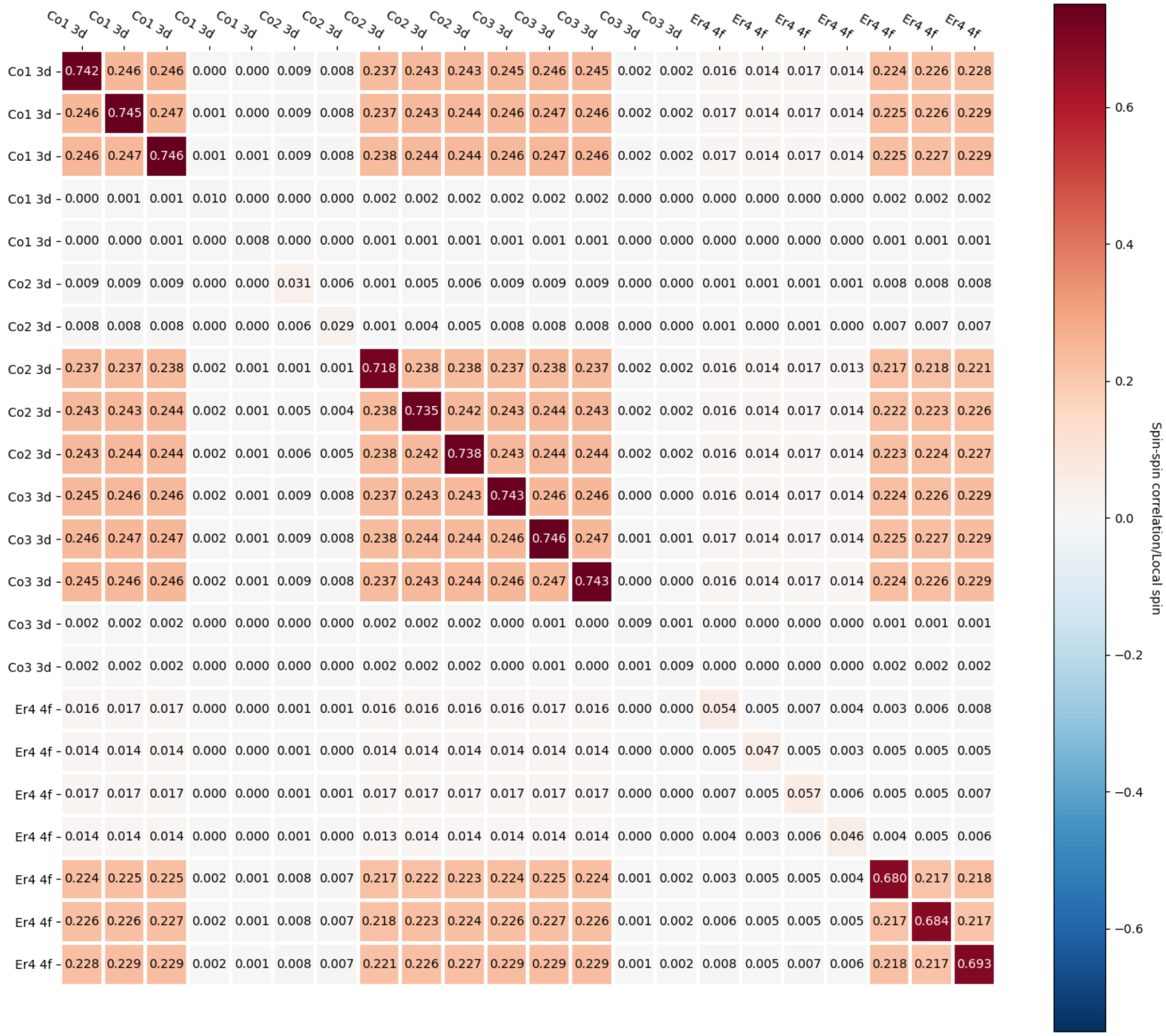

(b)

Figure S1: Local spin and spin-spin correlation of/between Co 3d and Er 4f orbitals in the FCIQMC(56,56) calculations. (a) and (b) show $S_{\text{tot}} = 0$ and $S_{\text{tot}} = 6$, respectively. The diagonal part of each subfigure provides the local spin of each orbitals and the off-diagonal part provides the spin-spin correlation between orbitals.

S3 DMRGCI input examples

In the following we show the input example for DMRGCI(56,56) M=1000 calculation for $S_{\mathrm{tot}} = 0$ state using BLOCK code.

```
nelec 56
spin   0
irrep  1
schedule
 0  250 0.0001 0.0001
 2  500 0.0001 0.0001
 4 1000 0.0001 0.0001
 6 1000 1e-05 1e-05
 8 1000 1e-06 1e-06
10 1000 1e-07 1e-07
12 1000 1e-08 0.0
end

maxiter 100
sweep_tol 1e-07
warmup local_4site
twodot_to_onedot 16
twopdm
orbitals FCIDUMP
symmetry c1
gaopt default
hf_occ 2 2 2 2 2 2 2 2 2 2 2 2 2 2 2 2 2 2 2 2 2 2 2 2 2 2 2 2 0 0 0 0 0 0 0 0 0 0 0 0 0 0 0 0 0 0 0 0 0 0 0 0 0 0 0 0
memory 100000 m
num_thrds 16
```

The same settings have been used for $S_{\mathrm{tot}} = 1 - 6$ states except for the keywords "spin" and "hf_occ". M=2000, 3000, 4000, 5000 calculations have been performed step-by-step from the previous calculation with the same settings. The sweep schedules are:

M=2000:

```
schedule
0 2000 1e-07 1e-07
2 2000 1e-08 0.0
end
```

M=3000:

```
schedule
0 3000 1e-07 1e-07
```

```
2 3000 1e-08 0.0
end
```

M=4000:

```
schedule
0 4000 1e-08 1e-08
2 4000 1e-09 0.0
end
```

M=5000:

```
schedule
0 5000 1e-09 1e-09
2 5000 1e-10 0.0
end
```

S4 FCIQMC input examples

In the following we show the input example for FCIQMC(56,56) 200×10^6 walkers calculation for $S_{\text{tot}} = 0$ state using NECI code.

```
Title

System read
    electrons  56
    nonuniformrandexcits mol_guga_weighted
    nobrillouintheorem
    guga 0
    freeformat
endsys

calc
    definedet     1    2    3    4    5    6    7    8    9   10   11   12   13   14   15   16   17   18   19   20   21   22   23   24   25   27   29   31   32   33   34   55   56   57   58   59   61   63   66   68   70   71   72   73   74   99  100  101  102  103  104  105  106  108  110  112

    methods
        method vertex fcimc
    endmethods

    totalwalkers    2.e8
    new-tau-search 0.9999 100000 20000
    truncate-spawns 2
```

```
    diagshift 0.00

    readpops
    walkcontgrow
    semi-stochastic 10000
    pops-core 10000

    shiftdamp 0.02
    nmcyc 200000
    stepsshift 10
    proje-changeref 1.2
    truncinitiator
    addtoinitiator  3
    allrealcoeff
    realspawncutoff 0.30
    jump-shift
    max-tau 0.02
    maxwalkerbloom 1
    memoryfacspawn 10.00
    memoryfacpart 5.00
    time 1400vim
    startsinglepart 100
endcalc
logging
    highlypopwrite 2000
    hdf5-pops
endlog
end
```

The input example for the corresponding RDM sampling is:

```
Title

System read
    electrons  56
    nonuniformrandexcits mol_guga_weighted
    nobrillouintheorem
    guga 0
    freeformat
endsys

calc
    definedet     1    2    3    4    5    6    7    8    9   10   11   12   13
       14   15   16   17   18   19   20   21   22   23   24   25   27   29   31
      32   33   34   55   56   57   58   59   61   63   66   68   70   71   72
     73   74   99  100  101  102  103  104  105  106  108  110  112

    methods
```

```
        method vertex fcimc
    endmethods

    totalwalkers    2.e8
    new-tau-search 0.9999 100000 20000
    truncate-spawns 2
    diagshift 0.00

    readpops
    semi-stochastic
    pops-core 10000

    shiftdamp 0.02
    nmcyc 200000
    stepsshift 10
    proje-changeref 1.2
    truncinitiator
    addtoinitiator  3
    allrealcoeff
    realspawncutoff 0.30
    jump-shift
    max-tau 0.02
    maxwalkerbloom 1
    memoryfacspawn 10.00
    memoryfacpart 5.00
    time 1400
    startsinglepart 100
    rdmsamplingiters 20000
endcalc
logging
    highlypopwrite 2000
    hdf5-pops
    write-spin-free-rdm
    printonerdm
    calcrdmonfly   3  0  1000
endlog
end
```

The same settings have been used for $S_{\text{tot}} = 1 - 6$ states except for the keywords "guga" and "definedet".
